# Characteristics of the Measurement Tools for Assessing Health Information–Seeking Behaviors in Nationally Representative Surveys: Systematic Review

**DOI:** 10.2196/27539

**Published:** 2021-07-26

**Authors:** Hanna Choi, Gyeonghui Jeong

**Affiliations:** 1 Department of Nursing Science Nambu University Gwangju Republic of Korea; 2 College of Nursing Chonnam National University Gwangju Republic of Korea; 3 College of Nursing Seoul National University Seoul Republic of Korea

**Keywords:** information seeking behavior, consumer health information, medical informatics, health care surveys, health information–seeking behavior, surveys

## Abstract

**Background:**

The coronavirus pandemic (COVID-19) has also emerged as an infodemic, thereby worsening the harm of the pandemic. This situation has highlighted the need for a deeply rooted understanding of the health information–seeking behaviors (HISBs) of people.

**Objective:**

The aim of this paper was to review and provide insight regarding methodologies and the construct of content in HISB surveys by answering the following research question: what are the characteristics of the measurement tools for assessing HISBs in nationally representative surveys around the world?

**Methods:**

The Preferred Reporting Items for Systematic Reviews and Meta-Analyses was used as the framework for this study. A data search was performed through 5 international and 2 Korean databases covering the years between 2008 and 2020. Initially, studies performed among nationally representative samples were included to discover HISB survey instruments. The methodologies of the studies using HISB surveys were analyzed. For content analysis, 2 researchers reached a consensus through discussion by scrutinizing the contents of each survey questionnaire.

**Results:**

A total of 13 survey tools from 8 countries were identified after a review of 2333 records from the search results. Five survey tools (Health Information National Trends Survey, Health Tracking Survey, Annenberg National Health Communication Survey, National Health Interview Survey, and Health Tracking Household Survey) from the United States, 2 instruments from Germany, and 1 tool from each of the countries of the European Union, France, Israel, Poland, South Korea, and Taiwan were identified. Telephone or web-based surveys were commonly used targeting the adult population (≥15 years of age). From the content analysis, the domains of the survey items were categorized as follows: information (information about health and patient medical records), channel (offline and online), and health (overall health, lifestyle, and cancer). All categories encompassed behavioral and attitude dimensions. A theoretical framework, that is, an information-channel-health structure for HISBs was proposed.

**Conclusions:**

The results of our study can contribute to the development and implementation of the survey tools for HISB with integrated questionnaire items. This will help in understanding HISB trends in national health care.

## Introduction

### Background

The recent global pandemic of COVID-19, determined to be a public health emergency of international concern, has changed many aspects of people’s daily lives [1]. When people wake up, they check health-related news, their signs and symptoms, methods of prevention, and restrictions on the use of a vaccine. While mass media have been releasing a myriad of information, individuals have also been reproducing and downloading news and information from internet webpages such as websites or blogs [2,3]. The tsunami of information has resulted in the production of several fake news that lack scientific evidence and convey misconceptions and misinformation about health [4]. In reality, misguided belief based on misinformation has caused the deaths of many people [5] and worsened COVID-19 infections [6,7]. In this way, the rise of incorrect information has led to abuse, or in other words, an infodemic [4,8]. The foremost solution to mitigate this issue would be to understand the information-seeking behaviors of individuals. It would be beneficial if governments or national institutes measure their behaviors to apply health and information policies appropriately [9].

Health information–seeking behavior (HISB) is a comprehensive term that describes an individual’s behavior of seeking information, including the intentional collection and unintentional receipt of information [10,11]. Some studies have shown HISBs by using certain measurement tools such as Health Information National Trends Survey (HINTS), Health Tracking Survey, and the Annenberg National Health Communication Survey (ANHCS). The limitations of these studies are that most surveys mainly target American subjects or web-based/digital HISB [12-18]. These limitations can be overcome by the design of a comprehensive survey instrument. Survey instruments are developed to collect information for certain research phenomena [19] or for finding the right answers by asking the right questions. It would be efficient and effective to obtain a holistic view by integrating the properties of worldwide national survey tools in a systematic approach and by scrutinizing the constructs and methodologies, including what aspects of HISBs are considered important or are missed out. Although there are preliminary studies using systematic reviews of HISB instruments, these topics are limited to the context of the United States and eHealth, thereby making it difficult to look into cross-national HISB [17,20]. Therefore, this study aims to review how HISBs are measured by identifying and comparing measurement tools based on nationwide surveys.

### Objectives

The aim of this paper was to provide insights on the methodologies and the construct of content for HISB survey instruments based on nationally representative surveys.

## Methods

### Research Question

The SPIDER (sample, phenomenon of interest, design, evaluation, and research type) format was used to formulate the research question for this review [21,22]: what are the characteristics of measurement tools (evaluation) for assessing HISBs (phenomenon of interest) in nationally representative surveys around the world (sample and design)?

### Protocol and Registration

This study was conducted in accordance with Preferred Reporting Items for Systematic Reviews and Meta-Analyses [23]. The protocol of this review paper is registered in PROSPERO (CRD42019122767).

### Eligibility Criteria

To answer the research question, inclusion and exclusion criteria were established. Survey tools were included if they were full versions of the tools for HISBs and if they targeted nationally representative samples. However, tools were excluded when the full versions of the instruments were not accessible, not HISB-focused, nor used for a nationally representative sample.

### Information Sources

**Figure 1:**
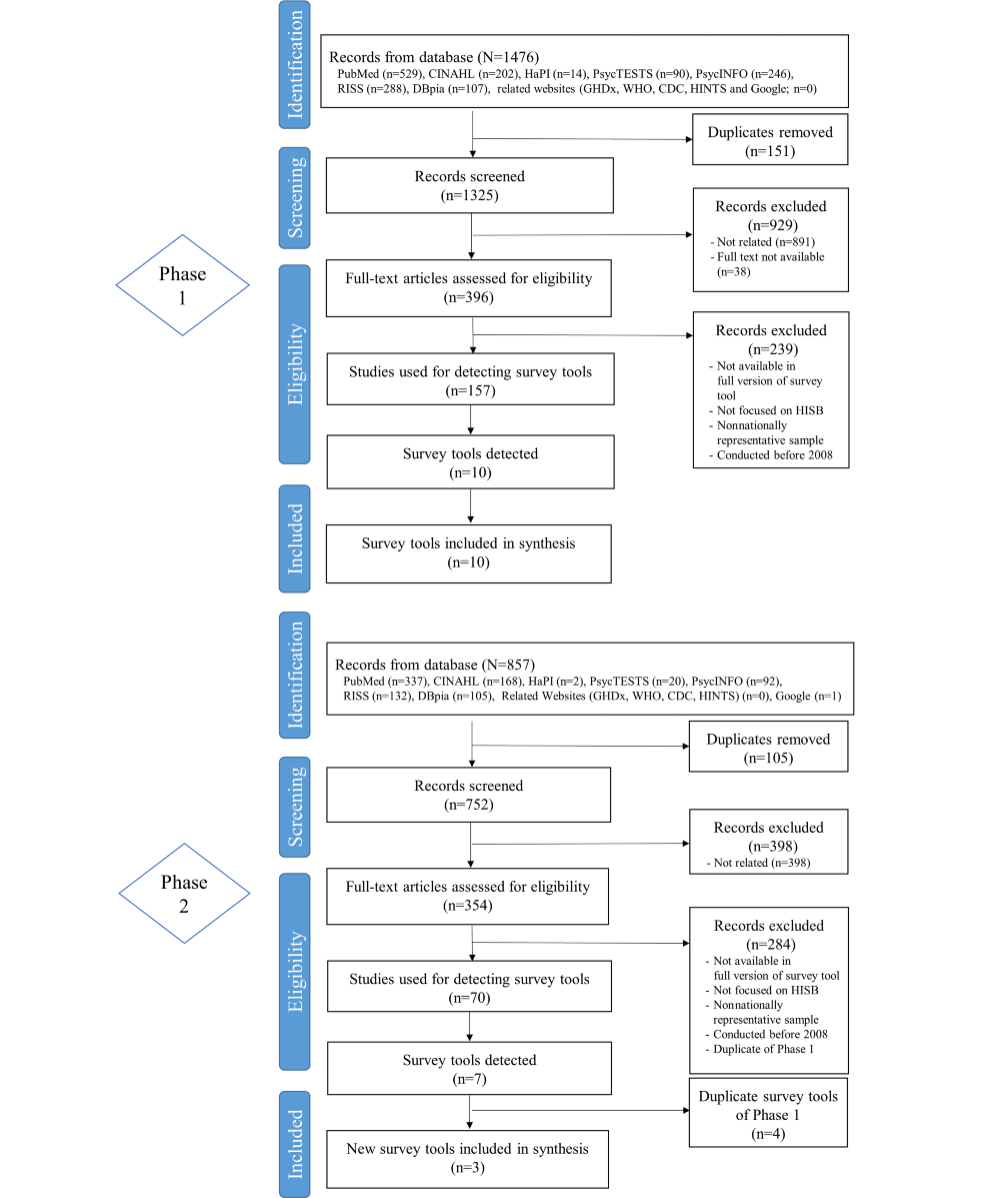
PRISMA flow diagram of literature search and selection process. CDC: Centers for Disease Control and Prevention; HINTS: Health Information National Trends Survey; HISB: health information–seeking behavior; RISS: Research Information Sharing Service; WHO: World Health Organization.

As we seek in this study to discover the national survey tools for HISB, articles, reports, and related websites were searched for clues to detect those instruments. The data search was performed in 2 phases. The phase 1 search covering 2008 to 2017 was conducted between October 09, 2017 and November 13, 2017 through 7 databases: 5 international databases, namely, PubMed, CINAHL Complete (Ebsco), HaPI, PsycTESTS, and PsycINFO (Ebsco), and 2 Korean databases (RISS [Research Information Sharing Service] and DBpia). Phase 2 was performed between February 19, 2021 and March 25, 2021 to obtain recent literature covering 2017 to 2020 with the same search strategy (Figure 1, Multimedia Appendix 1).

### Search Strategy

Pilot searches were performed by the authors, and the final search strategy with the consultation of a librarian was utilized with MeSH terms (ie, information-seeking behavior) and free-text searching as well as the Boolean operators “OR” and “AND” (Multimedia Appendix 1). There was no limit on languages, but publication years were restricted between 2008 and 2020: January 1, 2008 to November 13, 2017 for phase 1 and January 1, 2017 to December 31, 2020 for phase 2.

### Study Selection and Data Collection Process

Two authors (HC and GJ) initially reviewed the titles and abstracts of the papers and eliminated irrelevant documents. Then, HC and GJ scrutinized full-texts and filtered them according to the inclusion/exclusion criteria. As the purpose of the study was to seek nationally representative surveys of HISB, related websites were also accessed, such as that of The World Health Organization, which has the primary role of directing and coordinating international health, and Global Health Data Exchange [24], which is the most comprehensive catalog of surveys, censuses, vital statistics, and other health-related data in the world. In addition, to obtain the survey questionnaires, websites such as those of the National Cancer Institute, Centers for Disease Control and Prevention, European Commission, and Santé Publique France were searched. Academic papers, reports, and webpages identified through the previous steps were reviewed to discover HISB survey tools. To attain sufficient data (ie, full version of the item(s) of the survey, methodology, etc), we emailed 8 corresponding authors of the papers: 2 of the corresponding authors sent full version of the survey instruments, which were not related to the HISB; 1 author refused to provide a full version of the survey instrument; and 5 authors did not respond. To capture grey literature, footnote tracing was performed along with a review of the related websites described above. All documents identified through this process were managed with EndNote X20.0 software (Clarivate Analytics). During the whole process, consensus was reached through discussion if there was disagreement between the authors.

### Data Items

We sought the characteristics of the selected instruments, including the name of the instrument, administrative institution, and funding sources, country, language, frequency of the survey, survey duration, sampling method, mode of survey administration, target population, total number of the population, and purpose of the measurement. In addition, the content of the survey instruments was scrutinized.

### Risk of Bias in Individual Studies

The aim of this study was to identify the measures used to analyze HISB in national surveys. Therefore, this review paper focuses on questionnaires in the national surveys on HISB and the risk of bias assessment is not applicable.

### Synthesis of Results

As this review is intended as content analysis, the authors thoroughly read the contents of the questionnaires of the selected HISB instruments. Themes emerged during this process as we used coding sheets with Excel and Word. The findings were provided through the process of reaching a consensus between the 2 authors on the coding sheets. Finally, the synthesized results were depicted in table and figure formats.

## Results

### Study Selection

A total of 2333 papers were identified through 2 phases of the search process. From phase 1 of the search, 1476 papers were identified in the following academic databases: PubMed (n=529), CINAHL (n=202), HaPI (n=14), PsycTESTS (n=90), PsycINFO (n=246), RISS (n=288), and DBpia (n=107). Duplicates (n=151) were removed and 929 papers were eliminated. A total of 396 full-texts were reviewed and 157 documents were used for detecting 10 survey tools: (1) HINTS [25], (2) Health Tracking Survey [26], (3) ANHCS [27] (n=5), (4) National Health Interview Survey (NHIS) [28], (5) Health Tracking Household Survey (HTHS) [29], (6) Flash Eurobarometer [30], (7) Baromètre Santé [31], (8) Gesundheitsmonitor [32], (9) Israeli survey [33], and (10) eHealth Consumer Trend Survey [34].

Phase 2 was performed to update the recent survey tools by using the same search strategy. As a result, 857 records were identified: PubMed (n=337), CINAHL (n=168), HaPI (n=2), PsycTESTS (n=20), PsycINFO (n=92), RISS (n=132), DBpia (n=105), and Google (n=1). Duplicates (n=105) were excluded, and 398 records were also removed after screening. The full texts of 354 papers were reviewed, and 70 records were used for detecting 7 survey tools. There were 4 duplicates of survey tools from phase 1. Therefore, 3 more survey tools, that is, Stiftung Gesundheitswissen (HINTS Germany) [35], survey of cancer and health-related information–seeking behavior (CHISB) for Koreans [36], and Taiwan Communication Survey [37] were also included for synthesis.

A total of 227 papers were related to the selected HISB instruments (Multimedia Appendix 2). About 96% of them (219/227) were related to 1 of the 5 US surveys: HINTS [25] (n=188), the Health Tracking Survey [26] (n=9), ANHCS [27] (n=7), NHIS [28] (n=11), and HTHS [29] (n=4). The remaining 8 studies identified 8 survey tools used in other parts of the world, that is, European Union (Flash Eurobarometer) [30] (n=1), France (Baromètre santé) [31] (n=1), Germany (Gesundheitsmonitor [32] [n=1] and HINTS Germany) [35] [n=1]), Israeli survey [33] (n=1), Poland (eHealth Consumer Trend Survey) [34] (n=1), South Korea (survey of CHISB) [36] (n=1), and Taiwan (Taiwan Communication Survey) [37] (n=1). Therefore, 13 survey instruments (Table 1, Multimedia Appendix 3) were included in this review [38-77].

**Table 1:** Brief characteristics of the instruments for measuring health information–seeking behaviors in nationally representative survey studies.

Country	Instrument	Survey version	Purpose of the measurement	Frequency	Target population	Total population in the survey (N)
USA	Health Information National Trends Survey (HINTS) [38-41]	2019, HINTS 5, Cycle 3	To investigate respondents’ access to and use of health information, including information technology to manage health and health information	Every few years (1-2 year cycle)	Civilian noninstitutionalized adults aged 18 years or older	5247
USA	Health Tracking Survey [42-50]	2012	To assess pursuit of health taking place within a widening network of both online and offline sources	Irregular	Adults aged 18 years or older	3014
USA	Annenberg National Health Communication Survey [18,51-56]	2012	To capture national trends related to health behavior and behavioral intentions to media exposure, health knowledge and beliefs, and policy preferences and beliefs	One-cycle survey	Adults aged 18 years or older	3692
USA	National Health Interview Survey [57-67]	2020	To monitor the health of the population through the collection and analysis of the data	Annual	Household	33,138^a^
USA	Health Tracking Household Survey [68-71]	2010	To inform health care decision makers about changes in the health care system and the influence	Irregular (2-5 year period)	Household	16,671 individuals (n=9165 Family Insurance Units)
Europe	Flash Eurobarometer 404 (European citizen’s digital health literacy) [72]	2014	To support increasing use of digital health care to help manage citizen’s own health	One-cycle survey	EU residents aged 15 years and older	26,566 (28 EU countries)
France	French Health Barometer (Baromètre santé) [73]	2017	To gain a better understanding of French health knowledge, attitudes, beliefs, and behaviors	Annual	Adults aged 18-75 years	15,635^b^
Germany	Gesundheitsmonitor [74]	2015	To assess health-related knowledge, attitudes, and behaviors	Annual	Adults aged 18-79 years	1598
Germany	HINTS Germany [75]	2019	To close the gap in important health-related information actions and systematical health records	Every few years (1-2 year cycle)	Adults aged 18-79 years	2902
Israel	Not titled survey [33]	2014	To measure eHealth literacy for others, including perceived outcome of internet use	One-cycle survey	Adult aged 21 years and older	819
Poland	eHealth Consumer Trend Survey 2012^c^ [76]	2012	To show the trends in the perceptions and preferences of Polish citizens regarding internet use and factors affecting their usage	Irregular	Adults aged 15-80+ years	1000
South Korea	Survey of cancer and health-related information–seeking behavior for Koreans [36]	2018	To capture national phenomena of cancer and health-related health information–seeking behavior of Koreans	One-cycle survey	Adults aged 18-65+ years	1012
Taiwan	Taiwan Communication Survey [77]	2016	To explore media use behaviors among the general public, including health, risk, and disaster communication	Annual	Adults aged 18 years and older	2098

^a^2019 sample size was reported. Data and report for 2020 will be published in fall 2021.

^b^French Health Barometer: the survey questionnaires were changed according to the survey years. The 2017 version of the survey contains health information–seeking behavior and is included in this study.

^c^eHealth consumer trend survey of 2012 was modified from the eHealth Consumer Trends Survey (2007), which was conducted in Denmark, Germany, Greece, Latvia, Norway, Poland, and Portugal in the World Health Organization/European eHealth Consumer Trends project [78,79].

### Key Characteristics of the Surveys

#### Country

HISB surveys were found in 8 countries (Table 1, Multimedia Appendix 3). The United States has 5 HISB surveys (HINTS, Health Tracking Survey, ANHCS, NHIS, and HTHS), and the other 7 countries or regions, namely, the European Union, France, Germany, Israel, Poland, South Korea, and Taiwan conduct surveys called Flash Eurobarometer, Baromètre santé, Gesundheitsmonitor, Israeli survey (not titled), the eHealth consumer trend survey, survey of CHISB for Koreans, and Taiwan Communication Survey, respectively.

#### Language

As the surveys focused on domestic people, official or national languages were used (Table 1, Multimedia Appendix 3). For instance, the HINTS from the United States used 2 versions of the survey: English and Spanish. The European Union also performed the survey using the mother tongue of the responders.

#### Instrument and Administration Institution

HISB surveys were administered by national, nonprofit, public institutions, or individual researchers (Table 1, Multimedia Appendix 3). Five instruments, that is, HINTS, Flash Eurobarometer, NHIS, Baromètre santé, and Taiwan Communication Survey, were developed and administered by national institutes, namely, the National Cancer Institute in the United States, the National Center for Health Statistics in the United States, the Directorate‑General for Communications Networks of the European Commission, the National Institute for Prevention and Health Education in France, and the Ministry of Science Technology in Taiwan, respectively. Four instruments were obtained from nonprofit institutions: the Pew Research Center (HINTS), the Center for Studying Health System Change (ceased operation in 2013) (HTHS), Bertelsmann Stiftung (Gesundheitsmonitor), and Gesundheitswissen and Hanover Center for Health Communication at the Institute for Journalism and Communication Research (HINTS Germany). A survey (ANHCS) was conducted by 2 public institutions, namely, the Annenberg Schools for Communication at the University of Pennsylvania and the University of Southern California. Individual researchers developed 3 survey tools: the Israeli survey, the eHealth Consumer Trend Survey (Poland), and the survey of CHISB for Koreans (South Korea), with the Israeli and South Korean studies funded by national institutes.

#### Frequency of the Survey

The frequency of the surveys was found to be annual, every few years, one time, or irregular (Table 1, Multimedia Appendix 3). The annual or every few years surveys were HINTS (United States), NHIS (United States), Baromètre santé (France), Gesundheitsmonitor (Germany), HINTS Germany (Germany), and Taiwan Communication Survey (Taiwan). The others, namely, the Health Tracking Survey (United States), ANHCS (United States), HTHS (United States), Flash Eurobarometer 404, the Israeli survey, survey of CHISB for Koreans (South Korea), and eHealth Consumer Trend Survey (Poland) have been conducted once or irregularly.

#### Sampling and Mode of Administration

The most common approach has been randomization, in particular, sampling with random digit dialing and then administration through a computer-assisted telephone interview (Table 1, Multimedia Appendix 3). In addition, for sampling, two-stage sampling (stratifying sample addresses and selecting 1 adult within each household) was often used. When web-based panels were used for random sampling, units or strata layers divided by the population group, geographical districts, size of the settlement, and the locality’s socioeconomic status were utilized to prevent clashes.

#### Population

The range of this study is restricted to researching tools used with adults (Table 1, Multimedia Appendix 3). The standard age of adulthood in each country varies from 15 years to 21 years. Mostly, adults are defined as people who are 18 years of age or older, but in Europe and Poland, those who are 15 years or older are considered part of the adult population. In Israel, people older than 21 years are considered adults.

#### Purpose

The purposes were similar among the measurements: to monitor the use of health information in accordance with the type of information technology such as online or offline (Table 1, Multimedia Appendix 3). However, the detailed outcome of the studies pursued was different. For instance, the Baromètre santé (France) aimed to discover knowledge, attitudes, and behaviors toward HISB; however, the ANHCS (United States) pursued HISB related to media exposure, health knowledge and beliefs, and policy preferences and beliefs.

### Content Analysis of the Instruments

**Figure 2:**
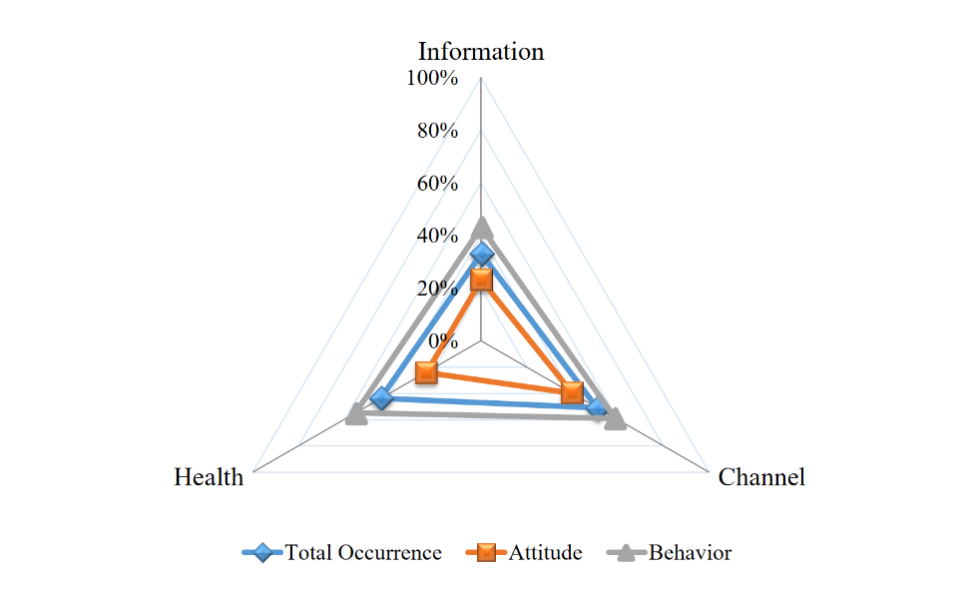
Average percentage of theme occurrence in the domains.

The contents of the questionnaire items for each tool were thematically reviewed and categorized by 2 researchers (HC and GJ). The themes were then merged and synthesized through consensus. Thus, 57 themes were detected and divided into 3 domains (Figure 2) and 7 subdomains: information, information about health and patient medical records; channel, offline and online; and health, overall health, lifestyle, and cancer. Two dimensions—attitude and behavior—were identified across the domains (Table 2, Multimedia Appendix 4). In this paper, attitude was defined as the emotional and cognitive tendency of a person toward a particular object, person, or thing, affecting behavior [80]. Behavior was also defined as an objectively observable activity [81].

**Table 2:** Content analysis of 13 representative national health information–seeking tools.^a^

Domain, subdomain, dimension, theme					Theme occurrence (%)		Theme occurrence average percentage (SD)		Subdomain average percentage (SD)			Domain average percentage (SD)	
**Information**													33.0 (14.9)
	**Information about health**									44.9 (14.9)			
		**Attitude**					26.9 (5.4)						
			Perceived ease of use	30.8									
			Perceived efficacy of seeking	23.1									
		**Behavior**					53.8 (6.3)						
			Search experience (frequency)	46.2									
			Information source	61.5									
			Type of information contents	53.8									
			Purpose of search (for whom)	53.8									
	**Patient medical record**									24.0 (6.4)			
		**Attitude**					21.5 (6.4)						
			Perceived privacy and confidentiality risk	23.1									
			Perceived ease of use	15.4									
			Perceived usefulness	23.1									
			Intention to use	15.4									
			Preference to provide access to others	30.8									
		**Behavior**					28.2 (4.4)						
			Access frequency	30.8									
			Type of information contents sought	23.1									
			Purpose of seeking a record	30.8									
**Channel**													50.5 (18.2)
	**Offline**									50.5 (15.9)			
		**Attitude**					41.0 (4.4)						
			Perceived credibility	38.5									
			Perceived ease of use	38.5									
			Satisfaction with service quality	46.2									
		**Behavior**					57.7 (18.3)						
			Access frequency	84.6									
			Type of health service	46.2									
			Communication with health care provider	46.2									
			Health-related decision making	53.8									
	**Online**									50.5 (19.7)			
		**Attitude**					39.6 (15.0)						
			Perceived credibility	53.8									
			Perceived ease of use	38.5									
			Perceived usefulness	53.8									
			Perceived eHealth literacy (technology efficacy)	53.8									
			Satisfaction with web-based information	15.4									
			Perceived confidentiality risks	30.8									
			Intention to use	30.8									
		**Behavior**					59.0 (19.2)						
			Access frequency	92.3									
			Type of information technology device	61.5									
			Health-related web and app (software use)	46.2									
			Web-based resource (governmental website, Wikipedia, etc)	53.8									
			Communication (consult) with health care provider	76.9									
			Communication with friends and others (social media, forum, etc)	61.5									
			Health-related decision making	69.2									
			Tracking/managing health state	38.5									
			Improvement of health knowledge	30.8									
**Health**													44.2 (20.6)
	**Overall health**									53.8 (18.0)			
		**Attitude**					34.6 (5.4)						
			Perceived health efficacy	38.5									
			Concerns and belief about health	30.8									
		**Behavior**					59.3 (16.4)						
			General health state	84.6									
			Diseases diagnosed	69.2									
			Height	61.5									
			Weight	61.5									
			Mental health	53.8									
			Caregiving	30.8									
			Social support	53.8									
	**Lifestyle**									32.7 (21.3)			
		**Attitude**					15.4 (8.9)						
			Perception about nutrition	7.7									
			Perception about physical activity	15.4									
			Perception about alcohol	23.1									
			Perception about tobacco	23.1									
		**Behavior**					50.0 (13.3)						
			Nutrition	30.8									
			Physical activity	61.5									
			Alcohol	53.8									
			Tobacco	53.8									
	**Cancer**									46.2 (13.3)			
		**Attitude**											
			Perception about cancer	38.5									
		**Behavior**					50.0 (16.3)						
			Cancer check-up	38.5									
			Cancer diagnosed	61.5									

^a^Total average percentage of the themes=44.0 (SD 19.3), total average percentage of attitude themes=30.4 (SD 13.5), and total average percentage of behavior themes=53.8 (SD 16.9).

#### Thematic Map

Three domains, namely, information, channel, and health (Figure 2) emerged through the content analysis (Table 2). The highest rate of theme occurrence among the domains was channel (average percentage 50.5%, SD 18.2), followed by health (average percentage 44.2%, SD 20.6) and information (average percentage 33.0%, SD 14.9).

##### Information

**Figure 3:**
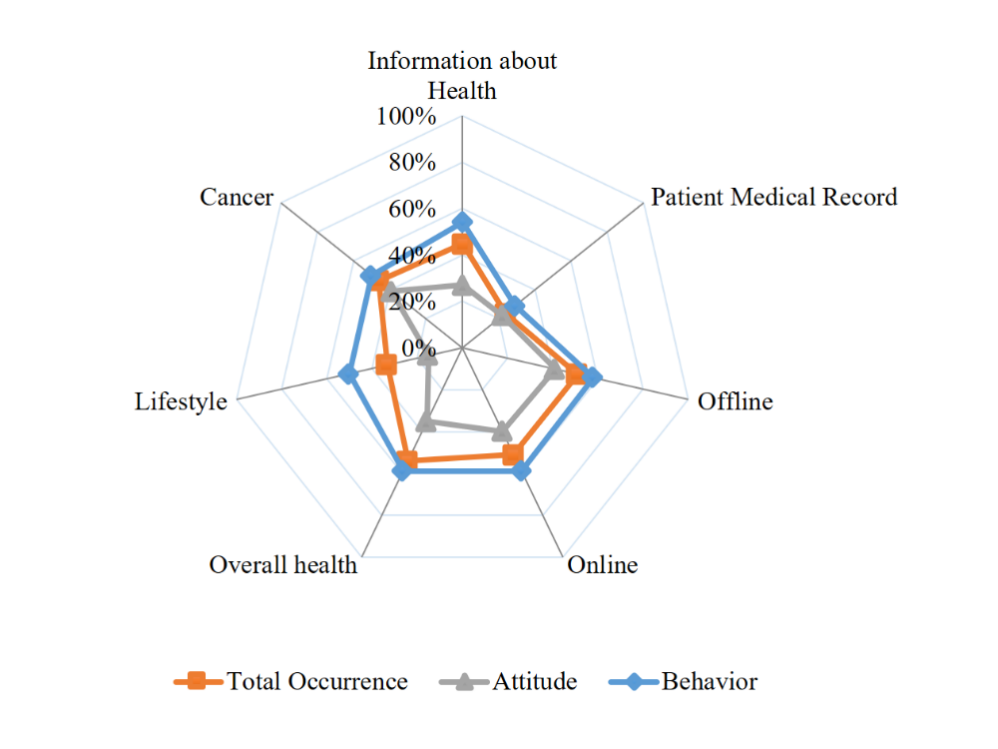
Average percentage of theme occurrence in the subdomains.

Information is a health-related, content-focused domain sought by the individual. There are 2 subdomains (Figure 3), namely, information about health and patient medical records. The information about the health subdomain was conceptualized by categorizing question items related to general health information through a set of options with comprehensive channels (online or offline). Patient medical records were related to a seeker’s use of medical records online or offline. There were attitude and behavioral aspects for the themes found, and the detailed and representative questionnaire items of the themes are presented in Table 3. The subdomain information about health (average percentage 44.9%, SD 14.9), which consisted of 6 themes, was more commonly used among the selected tools than patient medical records (average percentage 24.0%, SD 6.4), which consisted of 8 themes. In both subdomains, the percentages of behavior-related themes was 1.3-2.0 times higher than those related to attitude.

**Table 3:** Representative sample questionnaire items for health information–seeking behavior survey instruments.

Domain, subdomain, dimension, theme					Questionnaire items		
**Information**							
	**Information about health**						
		**Attitude**					
			Perceived ease of use	How much do you agree or disagree- it took a lot of effort to get the information you needed (HINTS^a^)			
			Perceived efficacy of seeking	How confident are you that you could get advice about health if you needed it (HINTS)			
		**Behavior**					
			Seek experience	Have you ever looked for information about health or medical topics from any source? (HINTS)			
			Information source	Thinking about the last time you had a serious health issue, did you get information from (selection of the information source)? (HTS^b^)			
			Type of information contents	What type of health-related information did you look for? (Europe)			
			Purpose of search (whom for)	The most recent time you looked for information about health or medical topics, who was it for? (HINTS)			
	**Patient medical record**						
		**Attitude**					
			Perceived privacy and confidentiality risk	Have you ever kept information from your health care provider because you were concerned about the privacy or security of your medical record? (HINTS)			
			Perceived ease of use	How easy or difficult was it to understand the health information in your online medical record? (HINTS)			
			Perceived usefulness	In general, how useful is your online medical record for monitoring your health? (HINTS)			
			Intention to use	Was denken Sie heute, werden Sie sich Ihre medizinischen Daten und Unterlagen mit Hilfe der Karte zukünftig näher anschauen? (What do you think today, will you take a closer look at your medical data and documents with the help of the card in the future?) (Gesundheitsmonitor, Germany)			
			Preference to provide access to others	In order to get a quick and valid diagnosis, I am positive about giving internet access to my medical record to a doctor in another location or abroad (Poland)			
		**Behavior**					
			Access frequency	Have you approached your family doctor, specialist, or other health professional(s) over the internet to read your health record? (Poland)			
			Type of information contents sought	귀하의 온라인 의료 기록에 다음과 같은 의료 정보가 포함되어 있습니까? (Do any of your online medical records include the following types of medical information?) (survey of CHISB^c^)			
			Purpose of seeking a record	In the past 12 months, have you used your web-based medical record to…(look up test results, monitor your health, etc) (HINTS).			
**Channel**							
	**Offline**						
		**Attitude**					
			Perceived credibility	Do you believe health-related information from medical staff at medical centers or pharmacies? (Taiwan)			
			Perceived ease of use	How difficult is it to contact a doctor or other health care providers at this place after their regular hours in case of urgent medical needs-very difficult, somewhat difficult, not too difficult, or not at all difficult? (HTHS)			
			Satisfaction with service quality	How satisfied are you with the health care you received in the past 12 months? (NHIS^d^)			
		**Behavior**					
			Access frequency	How many times have you personally been to the doctor within the last 12 months (Europe)			
			Type of health service	What kind of place do you go to most often - a clinic, doctor's office, emergency room, or some other place? (NHIS)			
			Communication with health care provider	In the past 12 months, did health care provider talk with you about all of the different prescription medicines you are using, including medicines prescribed by other doctors? (HTHS^e^)			
			Health related decision making	The following questions are about your communication with all doctors, nurses, or other health professionals you saw during the past 12 months: did they involve you in decisions about your health care as much as you wanted (HINTS)?			
	**Online**						
		**Attitude**					
			Perceived credibility	Selon vous, l’information de santé que vous avez obtenue la dernière fois est-elle crédible? (In your opinion, is the health information credible you obtained the last time (on the internet?) (France)			
			Perceived ease of use	In general, how comfortable do you feel. (using computers, internet, etc) (ANHCS^f^)			
			Perceived usefulness	How useful was the health information you found online? (HTHS)			
			Perceived eHealth literacy	I know how to use the internet to answer my health questions (Israel)			
			Satisfaction with web-based information	Overall, how satisfied or not are you with the health-related information you found on the internet? (Europe)			
			Perceived confidentiality risks	There are different reasons for not approaching your family doctor, specialist, or other health professional(s) via the internet. Which reasons apply to you? (I worry about confidentiality) (Poland)			
			Intention to use	Next time you want to get information on health-related questions, how likely are you to use the internet? (Europe)			
		**Behavior**					
			Access frequency	Within the last 12 months, have you used the internet to search for health-related information? (Europe)			
			Type of information technology device	Please indicate if you have each of the following: tablet computer like an iPad, smartphone, etc? (HINTS)			
			Health-related web and app (software use)	What kind of health apps do you currently have on your phone? (HTS)			
			Web-based resource (governmental website, Wikipedia, etc)	Have you used any of the following internet resources for health information? (government websites, news sites, etc) (ANHCS)			
			Communication with health care provider	Haben Sie diese Gesundheits-Apps auf Ihrem Tablet oder Smartphone schon einmal dazu genutzt, … um auf Gespräche mit Ihrem Arzt, Heilpraktiker, Physiotherapeuten usw. besser vorbereitet zu sein? (Have you ever used these health apps on your tablet or smartphone...to be better prepared for discussions with your doctor, alternative practitioner, physiotherapist, etc? (HINTS Germany)			
			Communication with friends and others (social media, forum, etc)	Still thinking just about the last 12 months, have you posted a health-related question online or shared your own personal health experience online in any way? (HTS)			
			Health-related decision making	Haben Sie diese Gesundheits-Apps auf Ihrem Tablet oder Smartphone schon einmal dazu genutzt, …. um zu entscheiden, wie mit einer Erkrankung umgegangen werden sollte? (Has your tablet or smartphone…helped you make a decision about how to treat an illness or condition? (HINTS Germany)			
			Tracking/managing health state	Has your tablet or smartphone helped you track progress on a health-related goal such as quitting smoking, losing weight, or increasing physical activity? (HINTS)			
			Improvement of health knowledge	Improved your understanding of the symptoms, conditions, or treatments in which you were interested (Israeli survey)			
**Health**							
	**Overall health**						
		**Attitude**					
			Perceived health efficacy	Overall, how confident are you about your ability to take good care of your health? (HINTS)			
			Concerns and belief about health	Agree that my good health is largely a matter of good fortune (ANHCS)			
		**Behavior**					
			General health state	How would you rate your level of health in general? (Europe)			
			Diseases diagnosed	Are you now living with any of the following health problems or conditions (diabetes, high blood pressure, etc) (HTS)			
			Height	How tall are you without shoes? (NHIS)			
			Weight	About how much do you weigh, in pounds, without shoes? (HINTS)			
			Mental health	Have you been diagnosed with any of the following medical conditions? (mental health condition) (ANHCS)			
			Caregiving	Are you a caregiver for an adult family member with any of the following medical conditions? (Alzheimer disease, cancer, etc) (ANHCS)			
			Social support	Is there anyone you can count on to provide you with emotional support when you need it, such as talking over problems or helping you make difficult decisions? (HINTS)			
	**Lifestyle**						
		**Attitude**					
			Perception about nutrition	How likely is it that eating 5 or more servings of fruits and vegetables every day will (make you look better)? (ANHCS)			
			Perception about physical activity	How likely is it that doing at least moderate exercise 3 or more times a week will (reduce your feelings of stress)? (ANHCS)			
			Perception about alcohol	How much do you agree or disagree with each of the following statements? (alcohol increases your risk of cancer) (HINTS)			
			Perception about tobacco	In your opinion, do you think that some smokeless tobacco products such as chewing tobacco, snus, and snuff are less harmful to a person's health than cigarettes? (HINTS)			
		**Behavior**					
			Nutrition	In the past week, on average, how many servings of fruit did you eat or drink per day? Please include 100% fruit juice, and fresh, frozen or canned fruits. (ANHCS)			
			Physical activity	In a typical week, how many days do you do any physical activity or exercise of at least moderate intensity, such as brisk walking, bicycling at a regular pace, and swimming at a regular pace? (HINTS)			
			Alcohol	In your entire life, have you had at least 12 drinks of any type of alcoholic beverage? (NHIS)			
			Tobacco	Have you smoked at least 100 cigarettes in your entire life? (ANHCS)			
	**Cancer**						
		**Attitude**					
			Perception about cancer	귀하께서는 다음 문항에 얼마나 동의하십니까? … 일상에서 접하는 모든 것이 암을 유발하는 원인임 (How much do you agree or disagree with each of the following statements? … It seems like everything causes cancer, There’s not much you can do to lower your chances of getting cancer, etc) (survey of CHISB)			
		**Behavior**					
			Cancer check-up	When did you have your most recent prostate-specific antigen test to check for prostate cancer? (ANHCS)			
			Cancer diagnosed	Have you ever been told by a doctor or other health professional that you had cancer or a malignancy of any kind? (NHIS)			

^a^HINTS: Health Information National Trends Survey.

^b^HTS: Health Tracking Survey.

^c^CHISB: cancer and health-related information–seeking behavior.

^d^NHIS: National Health Interview Survey.

^e^HTHS: Health Tracking Household Survey.

^f^ANHCS: Annenberg National Health Communication Survey.

##### Channel

The channel can be defined as the means-focused domain that enables seekers to acquire and transmit health information [50]. The contents of the questionnaires pointed out that there were 2 channels for HISB: offline and online. The offline channel includes any method that collects or transmits health information through non–web-based sources such as health care providers, books, magazines, friends, seminars, or other means, and the offline subdomain consists of 7 themes (Figure 3). The online channel refers to seeking health information via the internet with any information technology device; the online subdomain showed the largest number of themes, that is, 7 attitude and 9 behavior themes. The subdomains offline and online revealed a similar occurrence, with average percentages at 50.5% (SD 15.9) and 50.5% (SD 19.7), respectively. In particular, the average percentage of a behavioral dimension of the online channel, namely, access frequency, was counted as 92.3% in the selected HISB tools as well as 84.6% of the access frequency theme in the offline subdomain. The occurrence of behavior dimensions was 1.4-1.5 times that of the attitude dimensions.

##### Health

The health domain refers to the seeker’s physical status and perceptions about health: overall health, lifestyle, and presence of cancer. Overall health refers to general health status, including physical, mental, and social health and concerns or beliefs about them. Lifestyle consists of 4 parts of a person’s behavior and attitude: nutrition, physical activity, alcohol consumption, and tobacco consumption. Cancer themes focused on check-up and diagnosis with cancer perceptions. Overall health was the most frequently found subdomain out of the 7 subdomains (average percentage 53.8%, SD 18.0). The other subdomains, namely, lifestyle and cancer, revealed an average percentage of 32.7% and 46.2% with SD 21.3 and SD 13.3, respectively (Figure 3). In particular, the average percentage of behavioral themes in overall health and general health state accounted for 84.6% in the selected HISB tools, while perceptions about nutrition and physical activity accounted for the smallest percentage at 7.7%. Similar to other domains, the occurrence of behavior dimensions on the domain was 1.3-3.3 times higher than those of attitude.

##### Person Characteristics

A person is the subject of HISB who seeks and utilizes information. A person’s characteristics may affect HISB. The main considered characteristics throughout the instruments were age, sex or gender, nationality, race, language, education, income, occupational status, marital status, health literacy, health insurance, the number of household members, households with internet access, and preference for online or offline channels.

#### Themes Addressed by the Tools

**Figure 4:**
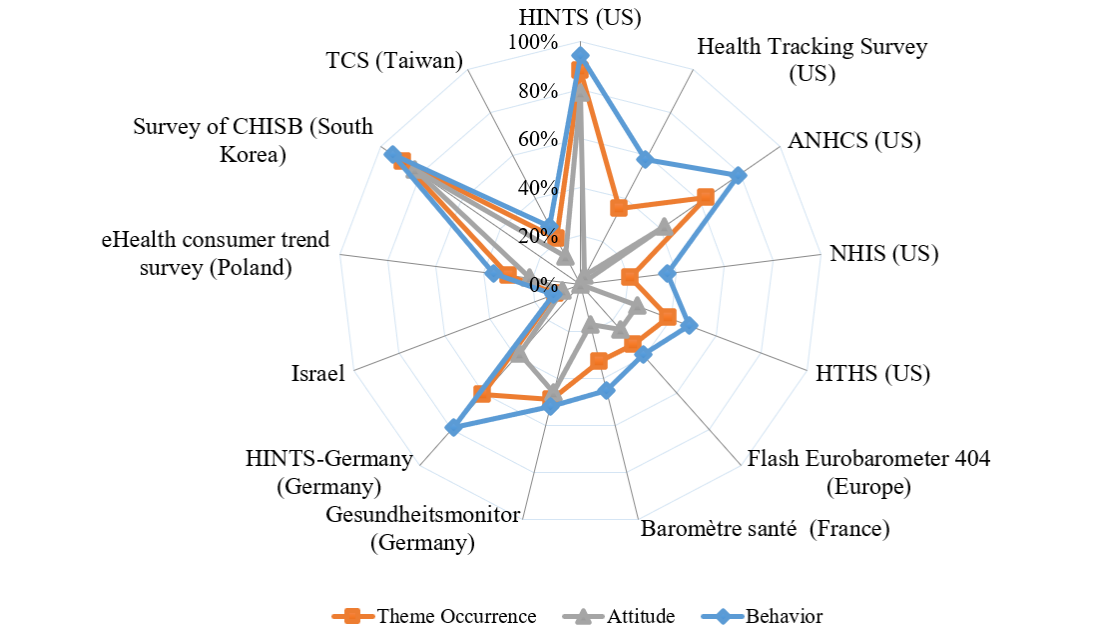
Average percentage of theme occurrence in health information–seeking behavior instruments. ANHCS: Annenberg National Health Communication Survey; HINTS: Health Information National Trends Survey; HISB: health information–seeking behavior; HTHS: Health Tracking Household Survey; NHIS: National Health Interview Survey; CHISB: cancer and health-related information–seeking behavior; TCS: Taiwan Communication Survey.

The spider web diagram shows the average percentage of the themes in the selected HISB tools. Survey of CHISB (South Korea) and HINTS (United States) accounted for 89% (51/57) and 88% (50/57), respectively, which were found to be high average percentages among the tools (Figure 4). ANHCS (United States), HINTS Germany, and Gesundheitsmonitor (Germany) also contain 63% (36/57), 61% (35/57), and 49% (28/57) of the contents of HISB, respectively. Other tools including the HTHS (United States), NHIS (United States), the Health Tracking Survey (United States), the Flash Eurobarometer (Europe), Baromètre santé (France), the eHealth Consumer Trend Survey (Poland), and Taiwan Communication Survey (Taiwan) showed similar percentages of 21%-39% (12-22 out of 57 themes). The other HISB measurement from Israel showed only 11% (6/57) of the contents. All the tools focused more, by far, on the behavioral dimension than on attitude, showing a total average percentage of 53.8% and 30.4%, respectively; moreover, each average percentage of the behavior dimension accounted for 1.2-14.5 times more than the attitude throughout the instruments.

#### Sample Questionnaire Items

From the content analysis, representative sample questionnaire items from the 13 survey instruments were selected. Table 3 presents each questionnaire according to the domains, subdomains, and themes with attitude and behavior dimensions.

### Proposed Theoretical Construct for Assessing HISB

**Figure 5:**
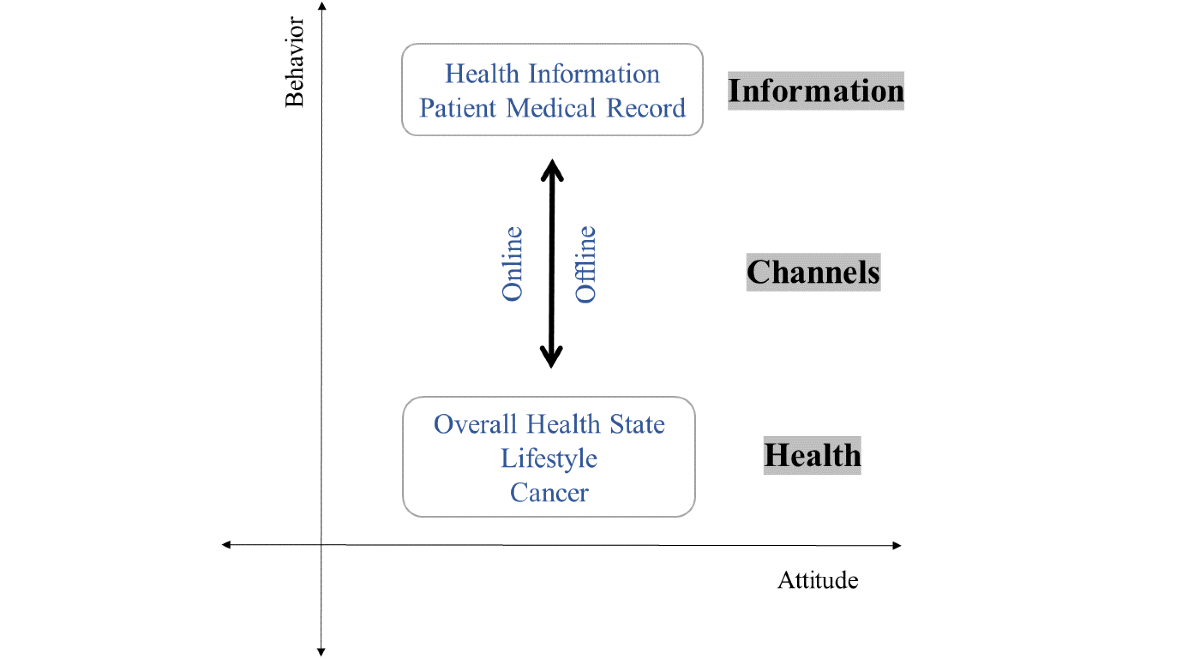
A proposed theoretical construct for health information–seeking behavior.

Through the content analysis, a theoretical framework emerged. This study proposed the information-channel-health structure for assessing HISB (Figure 5). The theoretical structure shows reciprocal interaction between information and health through channels within the attitude and behavior dimensions. The information-channel-health concepts include the following: information, with information about health and patient medical records; channels, as online and offline; and health, with overall health state, lifestyle, and cancer. With the reciprocal structure of information-channel-health underlying 2 dimensions (attitude and behavior), the HISB phenomenon could be well illustrated with a comprehensive and holistic view.

## Discussion

### Principal Results

In this study, we investigated the main characteristics of the methodologies and the contents of the HISB survey tools used for over more than a decade (2008-2020) to answer the following research question: what are the characteristics of the measurement tools for assessing HISBs in nationally representative surveys around the world? The aim of this paper is to provide insights on the methodologies and the construct of content for HISB survey instruments from nationally representative studies. Through the systematic search, 13 survey tools were found in 2333 records related to HISB surveys. The features of this study’s results are comprehensive and not limited to specific countries and specific topics or issue-based research. Other HISB-related review studies reported specific data such as age, college enrollment, adulthood, needs, and disease, including adolescent disease [82-84]. However, in this study, the results of the analysis were based on a tool for surveying healthy adults, who account for the highest proportion of the population density. Such a tool can lead to changes in the national policy.

The United States was found to have the most influential survey; 5 out of 13 tools developed in various countries were included in this study, a total of 188 research papers used data from HINTS, and HINTS identified 88% (50/57 themes) of the constructs, according to the findings. These strong features might be related to the purpose of HINTS to investigate respondents’ access to and use of health information, including information technology to manage health and health information. The composition of most of the questionnaire tools was continuously updated according to the change of the cycle. However, in the current survey of HINTS 5 Cycle 4, researchers changed its scope to focus on cancer compared to prior HINTS surveys, which focused on health and medical topics. Therefore, HINTS 5 Cycle 3 was included for the contents analysis part of this study. In particular, owing to the influence of COVID-19, the questionnaire in France was changed twice in 2020 only to reduce the time of survey completion.

The contents of each country’s survey tools contain the construct of HISBs. They can be categorized as information (information about health and patient medical records), channel (online and offline), and health (overall health state, lifestyle, and cancer), with dimensions of behavior and attitude. The questions are organized with more of the behavior dimension (average percentage of 53.8%) than attitude (average percentage of 30.4%) (Table 2). The analysis of the survey questionnaire contents conceptualized the HISB phenomenon, showing 3 domains, namely, information, channel, and health, with 2 dimensions, namely, behavior (objective outcome) and attitude (subjective tendency), emerging from the information-channel-health structure (Figure 5). In recent years, research has been conducted in parallel with existing reviews and meta-analysis to bring a theoretical framework to make some corrections [20] or to compare only specific variables to analyze the relationships [85] deductively. This study is meaningful as it derives a theoretical framework inductively after analyzing the contents by reviewing all the items of survey questionnaires. The findings of this study revealed that nationally representative surveys of HISB did not report theoretical frameworks when constructing the questionnaires. Therefore, it is believed that the outcomes of this study can be helpful in developing HISB-related tools or in establishing a theoretical framework prior to a large-scale investigation. This study included comprehensive (online and offline) HISBs. Recently, the terms eHealth and mobile health have become popular as many people use the internet and mobile access to manage their health. Therefore, preliminary review studies have focused on web-based HISBs or eHealth [17]. This research trend has a limitation in that it fails to address offline sources or face-to-face HISBs that still account for a large portion of HISBs.

This study found that all the survey instruments were from high-income countries, that is, United States, European Union, France, Germany, Israel, Poland, South Korea, and Taiwan, of the Organization for Economic Co-operation and Development [86]. The results can be interpreted as showing that there is information inequality, which may lead to a worsening of health inequality between high-income and low- and-middle-income countries. While low- and-middle-income countries still prioritize the establishment of universal health coverage focusing on the provider, high-income countries acknowledge the health information for individuals, empowering the health care consumer. The gap might be overcome through assessment of the trend of HISB in low- and-middle-income countries to contribute to the effective and efficient health care service to be provided. The details were analyzed by reviewing individual questions for the 13 survey tools, which were deeply rooted in the countries’ differences. There are deviations in the questions according to the culture or medical system. For example, the question options vary depending on whether the countries are exposed to terrorism or have specific diseases or causes of cancer such as ultraviolet radiation exposure followed by a high incidence of skin cancer. In addition, questions about the type of health insurance and Medicare system also varied—for example, whether to visit in-store retail clinics, where to receive prescriptions, differences in the quality of and satisfaction with medical services, and accessibility to medical services.

The degree of information technology development in the country also has a great influence on the questions. The question asking whether the respondent has computers or mobile/smart devices depends on the development of information technology and the retention rate of mobile phones in each country. As an extension of this question, questions were subdivided into digital literacy, the type of fitness app, and whether web-based chat groups were used for health-related topics. With HINTS as a standard, related studies from Germany, South Korea, and China were also developed. HINTS Germany was established by HINTS (United States) and supported by the National Cancer Institute. In the case of South Korea, an individual researcher developed the survey questionnaires based on the content of HINTS and was funded by a national institute. HINTS China was excluded in this study because researchers did not conduct a nationally representative sample survey of the country. These studies would enable cross-national trend analysis and agenda for HISB.

### Limitations and Recommendations for Future Research

For this study, we used databases in English and Korean, but there are some survey instruments that are neither English nor Korean. To overcome this limitation, we did not limit the languages in the search process. Moreover, it is obvious that English is the universal language of publication in the research field in the era of globalization. Therefore, we also used surveys in other languages, including 1 from France (French), Germany (German), and South Korea (Korean) in this paper. Some full versions of HISB survey instruments were not available for the review process. To attain the instrument, the researchers emailed corresponding authors for the HISB survey tools; however, these were found to be not related to HISB, or the author refused to provide a full version, or we received no response. In addition, the duration of the literature search was restricted to the period between 2008 and 2020. However, we mitigated this limitation because this study’s findings cover the fundamental essence of HISB phenomena by analyzing existing tools over a more extensive period. The theoretical framework derived from this study could be used as a guide for nationally representative HISB surveys. From the findings of this study, we see that there was a lack of theoretical basis for the survey instrument. The framework including both the behavior/attitude and online/offline dimensions would provide integrative scope for national HISB phenomena. Moreover, this framework could be compared to other HISB-related theories, thereby enabling more comprehensive insight into the HISB phenomenon. As the study scope focused on HISB instruments that seek nationally representative samples, future studies could also analyze different populations, including certain regions, ages, genders, and occupations with HISB instruments. It would be worthy to compare the differences among the populations.

### Conclusion

This study analyzed and synthesized current HISB survey questionnaires for nationally representative surveys. The findings of the methodology and content analysis provide a map and prototype for developing HISB-related instruments. A theoretical framework including both behavior/attitude and online/offline dimensions may provide integrative insight into real-world HISB phenomena. In sum, the findings of this study may contribute to better understanding of comprehensive HISB trends in nationally representative surveys.

## Appendix

Multimedia Appendix 1 Search strategies used for the study.

Multimedia Appendix 2 List of studies related to each health information–seeking behavior survey instrument tool.

Multimedia Appendix 3 Characteristics of the health information–seeking behavior survey instruments.

Multimedia Appendix 4 Theme occurrence table.

## Abbreviations

ANHCS: Annenberg National Health Communication Survey
CHISB: cancer and health-related information–seeking behavior
HINTS: Health Information National Trends Survey
HISB: health information–seeking behavior
HTHS: Health Tracking Household Survey
NHIS: National Health Interview Survey
RISS: Research Information Sharing Service
