# Supplementary material for: Characteristics of the Measurement Tools for Assessing Health Information–Seeking Behaviors in Nationally Representative Surveys: Systematic Review
Source: J Med Internet Res. 2021 Jul 26;23(7):e27539. doi: 10.2196/27539 (PMC8367171; doi:10.2196/27539)
Supplement: Multimedia Appendix 1 [file jmir_v23i7e27539_app1.pdf]

Multimedia Appendix 1. Search Strategies Used for the Study

1. PubMed

Set #	Search Terms	Results	
		Phase 1	Phase 2
1	(“information seeking behavior”[mesh] AND ("Consumer Health Information"[mesh] OR health[tw] OR patient*[tw]))<br>OR<br>(("Consumer Health Information"[mesh] OR "health information"[tw] OR "patient information"[tw]) AND (seek[tw] OR seeks[tw] OR sought[tw] OR seeking[tw]))	3595	5740
2	“Health Care Surveys”[mesh] OR “Health Surveys”[mesh] OR “Nutrition Surveys”[mesh] OR ((“Surveys and Questionnaires"[Mesh] OR survey[tw] OR surveys[tw] OR questionnaire[tw] OR questionnaires[tw]) AND (national[tw] OR nation[tw] OR nationwide[tw] OR nation-wide[tw] OR country-wide[tw] OR countrywide[tw] OR nationally[tw] OR representative[tw] OR population-based[tw]))	629,722	792,534
3	#1 AND #2	615	925
4	Phase 1: Filter (publish date) ("2008/01/01" - "2017/12/31")<br>Phase 2: Filter (publish date) 2017.01.01-2020.12.31	529	337

Phase 1: Search performed on November 13, 2017,
Phase 2: Search performed on February 18, 2021

2. Database: CINAHL Complete (Ebsco)

Set #	Search Terms	Results	
		Phase 1	Phase 2
**1**	(MH "Information Seeking Behavior" AND (MH "Health Information+" OR TX (health OR patient*))) OR (TI ((health N5 Information) N5 (seek OR seeks OR sought OR seeking)) OR AB ((health N5 Information) N5 (seek OR seeks OR sought OR seeking))) OR (TI ((patient N5 Information) N5 (seek OR seeks OR sought OR seeking)) OR AB ((patient N5 Information) N5 (seek OR seeks OR sought OR seeking))) OR (MH "Health Information+" AND TW (seek OR seeks OR sought OR seeking))	2783	4146
**2**	MH "Population Surveillance+" OR ((MH "Surveys+" OR MH "Questionnaires+" OR TX (survey OR surveys or questionnaire OR questionnaires)) AND TX (national OR nation OR nationwide OR nation-wide OR country-wide OR countrywide OR nationally OR representative OR population-based))	348,972	383,749
**3**	#1 AND #2	608	804
**4**	IN (Information N5 (seek OR seeks OR sought OR seeking)	12	16
**5**	#3 OR #4	618	817
**6**	Phase 1: #5 AND limit to Published Date: 20080101-20171231; Exclude MEDLINE records<br>Phase 2: #5 AND limit to Published Date: 2017.01-2020.12; Exclude MEDLINE records	202	168

Phase 1: Search performed on October 09, 2017
Phase 2: Search performed on February 18, 2021

3. Database: Health and Psychosocial Instruments (HaPI)

Set #	Search Terms	Results	
		Phase 1	Phase 2
**1**	("information seeking" AND ((health OR medical OR wellness OR patient))<br><br>Phase 1: 2008-2017.11.13(current)<br>Phase 2: 2017-2020	14	2

Phase 1: Search performed on November 13, 2017
Phase 2: Search performed on February 18, 2021

4. Database: PsycTESTS

Set #	Search Terms	Results	
		Phase 1	Phase 2
**1**	"information seeking" AND ((health OR medical OR wellness OR patient))<br>Phase 1: 2008-2017.11.13(current)<br>Phase 2: 2017-2020	90	20

Phase 1: Search performed on November 13, 2017
Phase 2: Search performed on February 18, 2021

5. Database: PsycINFO (Ebsco)

Set #	Search Terms	Results	
		Phase 1	Phase 2
**1**	DE "Information Seeking" OR TX ("health information seeking")	3664	4091
**2**	MA “Health Care Surveys” OR MA “Health Surveys” OR MA “Nutrition Surveys” OR ((DE "Surveys" OR DE "Consumer Surveys" OR DE "Mail Surveys" OR DE "Telephone Surveys" OR DE "Questionnaires" OR DE "General Health Questionnaire" OR TX (survey OR surveys or questionnaire OR questionnaires)) AND TX (national OR nation OR nationwide OR nation-wide OR country-wide OR countrywide OR nationally OR representative OR population-based))	117,049	140,738
**3**	#1 AND #2	293	362
**4**	TM ("information seeking" AND (health OR medical OR wellness OR patient))	33	46
**5**	#3 OR #4	320	398
**6**	Phase 1: #5 AND limit to Published Date: 20080101-20171231<br>Phase 2: #5 AND limit to Published Date: 2017.01-2020.12	246	92

Phase 1: Search performed on November 13, 2017
Phase 2: Search performed on February 18, 2021

6. Database: RISS

Set #	Search Terms	Results	
		Phase 1	Phase 2
1	건강정보추구(2,412) | 의료정보추구(944)	19,595	2931
2	도구	292,837	357,284
3	#1 AND #2	518	650
4	Phase 1: #3 AND (2008-2017)<br>Phase 2: #3 AND (2017-2020)	288	132

Phase 1: Search performed on October 22, 2017
Phase 2: Search performed on March 25, 2021

7. Database: DBpia*

Set #	Search terms	Result (phase 1)
1	TC='건강|의료' and TC='정보|추구'	3391
2	TC=’도구’	16,988
3	#1 AND #2	142
4	2008-2017	107

Set #	Search terms	Result (Phase 2)
1	(건강|의료) (정보|추구)	10,093
2	도구	31,547
3	#1 AND #2	374
4	2017-2020	105

* DBpia changed the search method, and it was applied to Phase 2.

Phase 1: Search performed on October 22, 2017
Phase 2: Search performed on March 25, 2021
