# Supplementary material for: Characteristics of the Measurement Tools for Assessing Health Information–Seeking Behaviors in Nationally Representative Surveys: Systematic Review
Source: J Med Internet Res. 2021 Jul 26;23(7):e27539. doi: 10.2196/27539 (PMC8367171; doi:10.2196/27539)
Supplement: Multimedia Appendix 2 [file jmir_v23i7e27539_app2.pdf]

Multimedia Appendix 2. Included studies

1. US_Health Information National Trends Survey (HINTS) (the National Cancer Institute (NCI))

1 Adjei Boakye E, Mohammed KA, Geneus CJ, Tobo BB, Wirth LS, Yang L, et al. Correlates of health information seeking between adults diagnosed with and without cancer. PLoS One. 2018;13(5):e0196446. PMID: 29746599. doi: 10.1371/journal.pone.0196446.

2 Adjei Boakye E, Mohammed KA, Osazuwa-Peters N, Lee MJ, Slomer L, Emuze D, et al. Palliative care knowledge, information sources, and beliefs: Results of a national survey of adults in the USA. Palliat Support Care. 2020 Jun;18(3):285-92. PMID: 31571557. doi: 10.1017/s1478951519000786.

3 Agurs-Collins T, Ferrer R, Ottenbacher A, Waters EA, O'Connell ME, Hamilton JG. Public Awareness of Direct-to-Consumer Genetic Tests: Findings from the 2013 U.S. Health Information National Trends Survey. J Cancer Educ. 2015 Dec;30(4):799-807. PMID: 25600375. doi: 10.1007/s13187-014-0784-x.

4 Alcalá HE, Sharif MZ, Morey BN. Misplaced Trust: Racial Differences in Use of Tobacco Products and Trust in Sources of Tobacco Health Information. Nicotine Tob Res. 2017 Oct 1;19(10):1199-208. PMID: 28387825. doi: 10.1093/ntr/ntx080.

5 Alhusseini N, Banta JE, Oh J, Montgomery S. Understanding the Use of Electronic Means to Seek Personal Health Information Among Adults in the United States. Cureus. 2020 Oct 27;12(10):e11190. PMID: 33269121. doi: 10.7759/cureus.11190.

6 Allen CG, McBride CM, Haardörfer R, Roberts MC. Associations Between Objective Television Exposure and Cancer Perceptions in a National Sample of Adults. Cancer Control. 2019 Jan-Dec;26(1):1073274819846603. PMID: 31131620. doi: 10.1177/1073274819846603.

7 Amo L. Education-based gaps in eHealth: A weighted logistic regression approach. J Med Internet Res. 2016;18(10):191-9. PMID: 2016-58576-015. doi: 10.2196/jmir.5188.

8 Amuta AO, Chen X, Mkuu R. The Effect of Cancer Information Seeking on Perceptions of Cancer Risks, Fatalism, and Worry Among a U.S. National Sample. American Journal of Health Education. 2017;48(6):366-73. PMID: 125829533. Language: English. Entry Date: 20171101. Revision Date: 20171101. Publication Type: Article. Journal Subset: Blind Peer Reviewed. doi: 10.1080/19325037.2017.1358119.

9 Andreeva VA, Unger JB, Yaroch AL, Cockburn MG, Baezconde-Garbanati L, Reynolds KD. Acculturation and sun-safe behaviors among US Latinos: findings from the 2005 Health Information National Trends Survey. Am J Public Health. 2009 Apr;99(4):734-41. PMID: 19150918. doi: 10.2105/ajph.2007.122796.

10 Arora NK, Hesse BW, Rimer BK, Viswanath K, Clayman ML, Croyle RT. Frustrated and confused: the American public rates its cancer-related information-seeking experiences. J Gen Intern Med. 2008 Mar;23(3):223-8. PMID: 17922166. doi: 10.1007/s11606-007-0406-y.

11 Atkinson NL, Saperstein SL, Pleis J. Using the internet for health-related activities: findings from a national probability sample. J Med Internet Res. 2009 Feb 20;11(1):e4. PMID: 19275980. doi: 10.2196/jmir.1035.

12 Bangerter LR, Griffin J, Harden K, Rutten LJ. Health Information-Seeking Behaviors of Family Caregivers: Analysis of the Health Information National Trends Survey. JMIR Aging. 2019 Jan 14;2(1):e11237. PMID: 31518309. doi: 10.2196/11237.

13 Barnes LLB, Khojasteh JJ, Wheeler D. Cancer information seeking and scanning: Sources and patterns. Health Educ J. 2017;76(7):853-68. PMID: 125907693. Language: English. Entry Date: 20171103. Revision Date: 20171103. Publication Type: Article. Journal Subset: Blind Peer Reviewed. doi: 10.1177/0017896917717542.

14 Barwise A, Cheville A, Wieland ML, Gajic O, Greenberg-Worisek AJ. Perceived knowledge of palliative care among immigrants to the United States: a secondary data analysis from the Health Information National Trends Survey. Ann Palliat Med. 2019 Sep;8(4):451-61. PMID: 30943736. doi: 10.21037/apm.2019.02.06.

15 Basch CH, MacLean SA, Romero R-A, Ethan D. Health Information Seeking Behavior Among College Students. Journal of Community Health. 2018;43(6):1094-9. PMID: 132789380. Language: English. Entry Date: 20181105. Revision Date: 20191202. Publication Type: Article. doi: 10.1007/s10900-018-0526-9.

16 Beckjord EB, Finney Rutten LJ, Arora NK, Moser RP, Hesse BW. Information processing and negative affect: evidence from the 2003 Health Information National Trends Survey. Health Psychol. 2008 Mar;27(2):249-57. PMID: 18377144. doi: 10.1037/0278-6133.27.2.249.

17 Benavidez G, Asare M, Lanning B, Ylitalo K, Fakhoury C, Thompson N, et al. Young adults' human papillomavirus–related knowledge: source of medical information matters. Public Health (Elsevier). 2020;182:125-30. PMID: 143415774. Language: English. Entry Date: 20200529. Revision Date: 20200529. Publication Type: Article. doi: 10.1016/j.puhe.2020.01.020.

18 Bernat JK, Ferrer RA, Margolis KA, Blake KD. US adult tobacco users' absolute harm perceptions of traditional and alternative tobacco products, information-seeking behaviors, and (mis)beliefs about chemicals in tobacco products. Addict Behav. 2017 Aug;71:38-45. PMID: 28259026. doi: 10.1016/j.addbeh.2017.02.027.

19 Bhuyan SS, Lu N, Chandak A, Kim H, Wyant D, Bhatt J, et al. Use of Mobile Health Applications for Health-Seeking Behavior Among US Adults. J Med Syst. 2016 Jun;40(6):153. PMID: 27147516. doi: 10.1007/s10916-016-0492-7.

20 Calixte R, Rivera A, Oridota O, Beauchamp W, Camacho-Rivera M. Social and Demographic Patterns of Health-Related Internet Use Among Adults in the United States: A Secondary Data Analysis of the Health Information National Trends Survey. Int J Environ Res Public Health. 2020 Sep 19;17(18). PMID: 32961766. doi: 10.3390/ijerph17186856.

21 Chae J. How we use the Internet matters for health: The relationship between various online health-related activities and preventive dietary behaviors. Health informatics journal. 2017 Oct 01:1460458217735675. PMID: 29047292. doi: 10.1177/1460458217735675.

22 Chan YM, Huang H. Weight Management Information Overload Challenges in 2007 HINTS: Socioeconomic, Health Status and Behaviors Correlates. Journal of Consumer Health on the Internet. 2013;17(2):151-67. PMID: 104172459. Language: English. Entry Date: 20130606. Revision Date: 20150820. Publication Type: Journal Article. doi: 10.1080/15398285.2013.780540.

23 Chen Y, Feeley TH. Numeracy, information seeking, and self-efficacy in managing health: an analysis using the 2007 Health Information National Trends Survey (HINTS). Health communication. 2014;29(9):843-53. PMID: 24266723. doi: 10.1080/10410236.2013.807904.

24 Cheng BT, Hauser JM. Adult palliative care in the USA: information-seeking behaviour patterns. BMJ Support Palliat Care. 2019 Nov 4. PMID: 31685523. doi: 10.1136/bmjspcare-2019-001928.

25 Cheng BT, Wangmo T, Hauser JM. Patterns of Palliative Care Beliefs Among Adults in the U.S.: Analysis of a National Cancer Database. J Pain Symptom Manage. 2019 Dec;58(6):1056-67. PMID: 31408666. doi: 10.1016/j.jpainsymman.2019.07.030.

26 Chou WY, Liu B, Post S, Hesse B. Health-related Internet use among cancer survivors: data from the Health Information National Trends Survey, 2003-2008. J Cancer Surviv. 2011 Sep;5(3):263-70. PMID: 21505861. doi: 10.1007/s11764-011-0179-5.

27 Chung JE. Patient-provider discussion of online health information: results from the 2007 Health Information National Trends Survey (HINTS). Journal of health communication. 2013;18(6):627-48. PMID: 23590202. doi: 10.1080/10810730.2012.743628.

28 Clayman ML, Manganello JA, Viswanath K, Hesse BW, Arora NK. Providing health messages to Hispanics/Latinos: Understanding the importance of language, trust in health information sources, and media use. Journal of health communication. 2010;15(Suppl 3):252-63. PMID: 2010-25748-020. doi: 10.1080/10810730.2010.522697.

29 Colon-Ramos U, Finney Rutten LJ, Moser RP, Colon-Lopez V, Ortiz AP, Yaroch AL. The association between fruit and vegetable intake, knowledge of the recommendations, and health information seeking within adults in the U.S. mainland and in Puerto Rico. Journal of health communication. 2015;20(1):105-11. PMID: 25204843. doi: 10.1080/10810730.2014.914607.

30 Cutrona SL, Mazor KM, Agunwamba AA, Valluri S, Wilson PM, Sadasivam RS, et al. Health Information Brokers in the General Population: An Analysis of the Health Information National Trends Survey 2013-2014. J Med Internet Res. 2016 Jun 03;18(6):e123. PMID: 27260952. doi: 10.2196/jmir.5447.

31 Cutrona SL, Mazor KM, Vieux SN, Luger TM, Volkman JE, Finney Rutten LJ. Health information-seeking on behalf of others: characteristics of "surrogate seekers". J Cancer Educ. 2015 Mar;30(1):12-9. PMID: 24989816. doi: 10.1007/s13187-014-0701-3.

32 Davis SN, O'Malley DM, Bator A, Ohman-Strickland P, Hudson SV. Correlates of Information Seeking Behaviors and Experiences Among Adult Cancer Survivors in the USA. J Cancer Educ. 2020 May 2. PMID: 32358717. doi: 10.1007/s13187-020-01758-6.

33 Donaldson EA, Hoffman AC, Zandberg I, Blake KD. Media exposure and tobacco product addiction beliefs: Findings from the 2015 Health Information National Trends Survey (HINTS-FDA 2015). Addict Behav. 2017 Sep;72:106-13. PMID: 28390232. doi: 10.1016/j.addbeh.2017.04.001.

34 Ellis EM, Ferrer RA, Klein WMP. Factors beyond Lack of Knowledge That Predict "I Don't Know" Responses to Surveys That Assess HPV Knowledge. J Health Commun. 2018;23(10-11):967-76. PMID: 30526402. doi: 10.1080/10810730.2018.1554729.

35 Emanuel AS, Godinho CA, Steinman C, Updegraff JA. Education differences in cancer fatalism: The role of information-seeking experiences. J Health Psychol. 2016 Aug 01:1359105316664129. PMID: 27553609. doi: 10.1177/1359105316664129.

36 Emanuel AS, Kiviniemi MT, Howell JL, Hay JL, Waters EA, Orom H, et al. Avoiding cancer risk information. Soc Sci Med. 2015 Dec;147:113-20. PMID: 26560410. doi: 10.1016/j.socscimed.2015.10.058.

37 Faith J, Thorburn S, Smit E. Body mass index and the use of the Internet for health information. Health Educ J. 2016;75(1):94-104. PMID: 112748177. Language: English. Entry Date: In Process. Revision Date: 20160912. Publication Type: Article. Journal Subset: Blind Peer Reviewed. doi: 10.1177/0017896914568435.

38 Faith J, Thorburn S, Tippens KM. Examining the association between patient-centered communication and provider avoidance, CAM use, and CAM-use disclosure. Altern Ther Health Med. 2015 Mar-Apr;21(2):30-5. PMID: 25830278.

39 Fareed N, Swoboda CM, Jonnalagadda P, Walker DM, Huerta TR. Differences Between Races in Health Information Seeking and Trust Over Time: Evidence From a Cross-Sectional, Pooled Analyses of HINTS Data. Am J Health Promot. 2021 Jan;35(1):84-92. PMID: 32588638. doi: 10.1177/0890117120934609.

40 Feirman SP, Donaldson EA, Parascandola M, Snyder K, Tworek C. Monitoring harm perceptions of smokeless tobacco products among U.S. adults: Health Information National Trends Survey 2012, 2014, 2015. Addict Behav. 2017 Sep 09;77:7-15. PMID: 28938110. doi: 10.1016/j.addbeh.2017.09.002.

41 Finney Rutten LJ, Agunwamba AA, Wilson P, Chawla N, Vieux S, Blanch-Hartigan D, et al. Cancer-Related Information Seeking Among Cancer Survivors: Trends Over a Decade (2003-2013). J Cancer Educ. 2016 Jun;31(2):348-57. PMID: 25712202. doi: 10.1007/s13187-015-0802-7.

42 Finney Rutten LJ, Blake KD, Greenberg-Worisek AJ, Allen SV, Moser RP, Hesse BW. Online Health Information Seeking Among US Adults: Measuring Progress Toward a Healthy People 2020 Objective. Public Health Rep. 2019 Nov/Dec;134(6):617-25. PMID: 31513756. doi: 10.1177/0033354919874074.

43 Finney Rutten LJ, Davis T, Beckjord EB, Blake K, Moser RP, Hesse BW. Picking up the pace: changes in method and frame for the health information national trends survey (2011-2014). Journal of health communication. 2012;17(8):979-89. PMID: 23020763. doi: 10.1080/10810730.2012.700998.

44 Finney Rutten LJ, Hesse BW, Moser RP, Ortiz Martinez AP, Kornfeld J, Vanderpool RC, et al. Socioeconomic and geographic disparities in health information seeking and Internet use in Puerto Rico. J Med Internet Res. 2012 Jul 19;14(4):e104. PMID: 22849971. doi: 10.2196/jmir.2007.

45 Fleary SA, Ettienne R. Inherited or Behavior? What Causal Beliefs about Obesity Are Associated with Weight Perceptions and Decisions to Lose Weight in a US Sample? International scholarly research notices. 2014;2014:632940. PMID: 27419204. doi: 10.1155/2014/632940.

46 Ford BM, Kaphingst KA. Lay interpersonal sources for health information related to beliefs about the modifiability of cancer risk. Cancer Causes Control. 2009;20(10):1975-83. PMID: 2009-21957-019. doi: 10.1007/s10552-009-9392-1.

47 Gerido LH, Tang X, Ernst B, Langford A, He Z. Patient Engagement in Medical Research Among Older Adults: Analysis of the Health Information National Trends Survey. J Med Internet Res. 2019 Oct 29;21(10):e15035. PMID: 31663860. doi: 10.2196/15035.

48 Giordano L, Leafman J, Citrin D, Wallace L. Cancer Information-Seeking Practices Among the Hispanic Population: Data From the Health Information National Trends Survey 2007. Hisp Health Care Int. 2015;13(2):70-6. PMID: 26078025. doi: 10.1891/1540-4153.13.2.70.

49 Go E, You KH. Health-related online information seeking and behavioral outcomes: Fatalism and self-efficacy as mediators. Social Behavior and Personality: An International Journal. 2018;46(5):871-9. PMID: 2018-31649-015. doi: 10.2224/sbp.6501.

50 Goldner M, Hale TM, Cotten SR, Stern MJ, Drentea P. The intersection of gender and place in online health activities. Journal of health communication. 2013;18(10):1235-55. PMID: 23886026. doi: 10.1080/10810730.2013.778364.

51 Greenberg AJ, Serrano KJ, Thai CL, Blake KD, Moser RP, Hesse BW, et al. Public use of electronic personal health information: Measuring progress of the Healthy People 2020 Objectives. Health policy and technology. 2017 Mar;6(1):33-9. PMID: 28480160. doi: 10.1016/j.hlpt.2016.08.003.

52 Gumataotao AP. Cancer information seeking on the internet: Disparities among adults with personal cancer experience. US: ProQuest Information & Learning; 2008.

53 Hale TM, Cotten SR, Drentea P, Goldner M. Rural-urban differences in general and health-related Internet use. Am Behav Sci. 2010;53(9):1304-25. PMID: 2010-08240-005. doi: 10.1177/0002764210361685.

54 Hale TM. Health status and health behavior as factors predicting online health seeking. US: ProQuest Information & Learning; 2012.

55 Hartoonian N, Ormseth SR, Hanson ER, Bantum EO, Owen JE. Information-seeking in cancer survivors: application of the Comprehensive Model of Information Seeking to HINTS 2007 data. Journal of health communication. 2014;19(11):1308-25. PMID: 24742287. doi: 10.1080/10810730.2013.872730.

56 Hay J, Coups EJ, Ford J, DiBonaventura M. Exposure to mass media health information, skin cancer beliefs, and sun protection behaviors in a United States probability sample. J Am Acad Dermatol. 2009 Nov;61(5):783-92. PMID: 19596487. doi: 10.1016/j.jaad.2009.04.023.

57 Hay JL, Gold GS, Baser RE, Hricak H, Dauer LT. Prevalence and Correlates of Worry About the Health Harms of Medical Imaging Radiation in the General Population. J Prim Care Community Health. 2016 Oct;7(4):219-25. PMID: 27162081. doi: 10.1177/2150131916648917.

58 Hesse BW, Arora NK, Burke Beckjord E, Finney Rutten LJ. Information support for cancer survivors. Cancer. 2008 Jun 01;112(11 Suppl):2529-40. PMID: 18428201. doi: 10.1002/cncr.23445.

59 Hesse BW, Moser RP, Rutten LJ. Surveys of physicians and electronic health information. N Engl J Med. 2010 Mar 04;362(9):859-60. PMID: 20200398. doi: 10.1056/NEJMc0909595.

60 Hoffman-Goetz L, Meissner HI, Thomson MD. Literacy and cancer anxiety as predictors of health status: an exploratory study. J Cancer Educ. 2009;24(3):218-24. PMID: 19526411. doi: 10.1080/08858190902910871.

61 Hong T. Internet health information in the patient-provider dialogue. Cyberpsychology & behavior : the impact of the Internet, multimedia and virtual reality on behavior and society. 2008 Oct;11(5):587-9. PMID: 18771392. doi: 10.1089/cpb.2007.0172.

62 Hong YA, Cho J. Has the Digital Health Divide Widened? Trends of Health-Related Internet Use Among Older Adults From 2003 to 2011. J Gerontol B Psychol Sci Soc Sci. 2017 Sep 01;72(5):856-63. PMID: 27558403. doi: 10.1093/geronb/gbw100.

63 Hong YA, Jinmyoung C. Has the Digital Health Divide Widened? Trends of Health-Related Internet Use Among Older Adults From 2003 to 2011. Journals of Gerontology Series B: Psychological Sciences & Social Sciences. 2017;72(5):856-63. PMID: 124650365. Language: English. Entry Date: 20170821. Revision Date: 20180903. Publication Type: Article. doi: 10.1093/geronb/gbw100.

64 Hou J, Shim M. The role of provider-patient communication and trust in online sources in Internet use for health-related activities. Journal of health communication. 2010;15 Suppl 3:186-99. PMID: 21154093. doi: 10.1080/10810730.2010.522691.

65 Huang H, Apouey B, Andrews J. Racial and Ethnic Disparities in Awareness of Cancer Genetic Testing Among Online Users: Internet Use, Health Knowledge, and Socio-Demographic Correlates. Journal of Consumer Health on the Internet. 2014;18(1):15-30. PMID: 104033331. Language: English. Entry Date: 20140304. Revision Date: 20150820. Publication Type: Journal Article. doi: 10.1080/15398285.2014.869165.

66 Huerta TR, Walker DM, Johnson T, Ford EW. A Time Series Analysis of Cancer-Related Information Seeking: Hints From the Health Information National Trends Survey (HINTS) 2003-2014. Journal of health communication. 2016 Sep;21(9):1031-8. PMID: 27565190. doi: 10.1080/10810730.2016.1204381.

67 Huerta TR, Walker DM, Mullen D, Johnson TJ, Ford EW. Trends in E-Cigarette Awareness and Perceived Harmfulness in the U.S. Am J Prev Med. 2017 Mar;52(3):339-46. PMID: 27890516. doi: 10.1016/j.amepre.2016.10.017.

68 Jabson JM, Patterson JG, Kamen C. Understanding Health Information Seeking on the Internet Among Sexual Minority People: Cross-Sectional Analysis From the Health Information National Trends Survey. JMIR public health and surveillance. 2017 Jun 19;3(2):e39. PMID: 28630036. doi: 10.2196/publichealth.7526.

69 Jackson I, Osaghae I, Ananaba N, Etuk A, Jackson N, Chido-Amajuoyi OG. Sources of health information among U.S. cancer survivors: results from the health information national trends survey (HINTS). AIMS Public Health. 2020;7(2):363-79. PMID: 32617363. doi: 10.3934/publichealth.2020031.

70 Jiang S, Beaudoin CE. Health literacy and the internet: An exploratory study on the 2013 HINTS survey. Comput Human Behav. 2016;58:240-8. PMID: 2016-12704-025. doi: 10.1016/j.chb.2016.01.007.

71 Jiang S, Liu PL. Digital divide and Internet health information seeking among cancer survivors: A trend analysis from 2011 to 2017. Psychooncology. 2020 Jan;29(1):61-7. PMID: 31652360. doi: 10.1002/pon.5247.

72 Jiang S, Street RL. Factors Influencing Communication with Doctors via the Internet: A Cross-Sectional Analysis of 2014 HINTS Survey. Health communication. 2017 Feb;32(2):180-8. PMID: 27196037. doi: 10.1080/10410236.2015.1110867.

73 Jiyoung C. How we use the Internet matters for health: The relationship between various online healthrelated activities and preventive dietary behaviors. Health Informatics Journal. 2019;25(3):973-83. PMID: 138627470. Language: English. Entry Date: 20190918. Revision Date: 20190918. Publication Type: Article. doi: 10.1177/1460458217735675.

74 Jo HS, Park K, Jung SM. A scoping review of consumer needs for cancer information. Patient Education and Counseling. 2019;102(7):1237-50. PMID: 2019-08801-001. doi: 10.1016/j.pec.2019.02.004.

75 Jun J, Kim SH, Wu L. Tobacco Risk Information and Comparative Risk Assessment of E-Cigarettes Vs. Cigarettes: Application of the Reinforcing Spirals Model. J Health Commun. 2019;24(4):422-31. PMID: 31210588. doi: 10.1080/10810730.2019.1630526.

76 Jun J, Nan X. Comparative risk assessment and cessation information seeking among smokeless tobacco users. Addict Behav. 2018 May;80:14-21. PMID: 29306721. doi: 10.1016/j.addbeh.2017.12.031.

77 Jun J. Cancer/health communication and breast/cervical cancer screening among Asian Americans and five Asian ethnic groups. Ethn Health. 2020 Oct;25(7):960-81. PMID: 29792075. doi: 10.1080/13557858.2018.1478952.

78 Kaufman A, Augustson E, Davis K, Finney Rutten LJ. Awareness and use of tobacco quitlines: evidence from the Health Information National Trends Survey. Journal of health communication. 2010;15 Suppl 3:264-78. PMID: 21154098. doi: 10.1080/10810730.2010.526172.

79 Kealey E, Berkman CS. The relationship between health information sources and mental models of cancer: findings from the 2005 Health Information National Trends Survey. Journal of health communication. 2010;15 Suppl 3:236-51. PMID: 21154096. doi: 10.1080/10810730.2010.522693.

80 Kim EJ, Yuan Y, Liebschutz J, Cabral H, Kazis L. Understanding the Digital Gap Among US Adults With Disability: Cross-Sectional Analysis of the Health Information National Trends Survey 2013. JMIR Rehabil Assist Technol. 2018 Mar 16;5(1):e3. PMID: 29549074. doi: 10.2196/rehab.8783.

81 Kim H, Chang CF. Effectiveness of Using Personal Health Records to Improve Recommended Breast Cancer Screening and Reduce Racial and Geographic Disparities Among Women. J Cancer Educ. 2020 Jul 9. PMID: 32648239. doi: 10.1007/s13187-020-01821-2.

82 Kim H, Filson C, Joski P, von Esenwein S, Lipscomb J. Association Between Online Information-Seeking and Adherence to Guidelines for Breast and Prostate Cancer Screening. Prev Chronic Dis. 2018 Apr 19;15:E45. PMID: 29679480. doi: 10.5888/pcd15.170147.

83 Kim H, Paige Powell M, Bhuyan S. Seeking Medical Information Using Mobile Apps and the Internet: Are Family Caregivers Different from the General Public? Journal of Medical Systems. 2017;41(3):1-8. PMID: 121441734. Language: English. Entry Date: 20170302. Revision Date: 20180530. Publication Type: Article. doi: 10.1007/s10916-017-0684-9.

84 Kim H, Paige Powell M, Bhuyan SS, Bhuyan SS. Seeking Medical Information Using Mobile Apps and the Internet: Are Family Caregivers Different from the General Public? J Med Syst. 2017 Mar;41(3):38. PMID: 28101781. doi: 10.1007/s10916-017-0684-9.

85 Kim K, Kwon N. Profile of e-patients: analysis of their cancer information-seeking from a national survey. Journal of health communication. 2010 Oct;15(7):712-33. PMID: 21104502. doi: 10.1080/10810730.2010.514031.

86 Knippen KL, Mahas R, Van Wasshenova E. Outcome Expectancies, Health Information Seeking, and Cancer Beliefs Associated with Multivitamin/Mineral Use in a National Sample, HINTS-FDA 2015. J Acad Nutr Diet. 2020 Aug;120(8):1368-76. PMID: 32061553. doi: 10.1016/j.jand.2019.12.008.

87 Kobayashi LC, Smith SG. Cancer Fatalism, Literacy, and Cancer Information Seeking in the American Public. Health Educ Behav. 2016 Aug;43(4):461-70. PMID: 26377524. doi: 10.1177/1090198115604616.

88 Koch-Weser S, Bradshaw YS, Gualtieri L, Gallagher SS. The Internet as a health information source: findings from the 2007 Health Information National Trends Survey and implications for health communication. Journal of health communication. 2010;15 Suppl 3:279-93. PMID: 21154099. doi: 10.1080/10810730.2010.522700.

89 Kontos E, Blake KD, Chou WY, Prestin A. Predictors of eHealth usage: insights on the digital divide from the Health Information National Trends Survey 2012. J Med Internet Res. 2014 Jul 16;16(7):e172. PMID: 25048379. doi: 10.2196/jmir.3117.

90 Kontos EZ, Emmons KM, Puleo E, Viswanath K. Contribution of communication inequalities to disparities in human papillomavirus vaccine awareness and knowledge. Am J Public Health. 2012 Oct;102(10):1911-20. PMID: 22970692. doi: 10.2105/ajph.2011.300435.

91 Kowalczyk N, Draper LJ. Trends in patient information preferences and acquisition. Radiol Technol. 2012 Mar-Apr;83(4):316-24. PMID: 22461341.

92 Kushalnagar P, Harris R, Paludneviciene R, Hoglind T. Health Information National Trends Survey in American Sign Language (HINTS-ASL): Protocol for the Cultural Adaptation and Linguistic Validation of a National Survey. JMIR research protocols. 2017 Sep 13;6(9):e172. PMID: 28903891. doi: 10.2196/resprot.8067.

93 Langford A, Loeb S. Perceived Patient-Provider Communication Quality and Sociodemographic Factors Associated With Watching Health-Related Videos on YouTube: A Cross-Sectional Analysis. J Med Internet Res. 2019 May 17;21(5):e13512. PMID: 31102372. doi: 10.2196/13512.

94 Langford A, Resnicow K, An L. Clinical trial awareness among racial/ethnic minorities in HINTS 2007: sociodemographic, attitudinal, and knowledge correlates. Journal of health communication. 2010;15 Suppl 3:92-101. PMID: 21154086. doi: 10.1080/10810730.2010.525296.

95 Langston ME, Fuzzell L, Lewis-Thames MW, Khan S, Moore JX. Disparities in Health Information-Seeking Behaviors and Fatalistic Views of Cancer by Sexual Orientation Identity: A Nationally Representative Study of Adults in the United States. LGBT Health. 2019 May/Jun;6(4):192-201. PMID: 31107153. doi: 10.1089/lgbt.2018.0112.

96 LaValley SA, Kiviniemi MT, Gage-Bouchard EA. Where people look for online health information. Health information and libraries journal. 2017 Jun;34(2):146-55. PMID: 27207817. doi: 10.1111/hir.12143.
97 Lee CJ, Chae J. An Initial Look at the Associations of a Variety of Health-Related Online Activities With Cancer Fatalism. Health communication. 2016 Nov;31(11):1375-84. PMID: 27007443. doi: 10.1080/10410236.2015.1072885.
98 Lee S. Predicting cancer information seeking behaviors of smokers, former smokers and nonsmokers using the 2012 Health Information National Trends Survey. US: ProQuest Information & Learning; 2014.
99 Lewis-Thames MW, Langston ME, Fuzzell L, Khan S, Moore JX, Han Y. Rural-urban differences e-cigarette ever use, the perception of harm, and e-cigarette information seeking behaviors among U.S. adults in a nationally representative study. Prev Med. 2020 Jan;130:105898. PMID: 31760117. doi: 10.1016/j.ypmed.2019.105898.
100 Lu L, Liu J, Yuan YC. Health Information Seeking Behaviors and Source Preferences between Chinese and U.S. Populations. J Health Commun. 2020 Jun 2;25(6):490-500. PMID: 33150861. doi: 10.1080/10810730.2020.1806414.
101 Lubetkin EI, Zabor EC, Isaac K, Brennessel D, Kemeny MM, Hay JL. Health literacy, information seeking, and trust in information in Haitians. Am J Health Behav. 2015 May;39(3):441-50. PMID: 25741688. doi: 10.5993/ajhb.39.3.16.
102 Lumpkins CY, Mabachi N, Lee J, Pacheco C, Greiner KA, Geana M. A Prescription for Internet Access: Appealing to Middle-Aged and Older Racial and Ethnic Minorities Through Social Network Sites to Combat Colorectal Cancer. Health communication. 2017 Jul;32(7):916-20. PMID: 27435103. doi: 10.1080/10410236.2016.1195679.
103 Lustria ML, Smith SA, Hinnant CC. Exploring digital divides: an examination of eHealth technology use in health information seeking, communication and personal health information management in the USA. Health informatics journal. 2011 Sep;17(3):224-43. PMID: 21937464. doi: 10.1177/1460458211414843.
104 Madadi M, Zhang S, Yeary KH, Henderson LM. Analyzing factors associated with women's attitudes and behaviors toward screening mammography using design-based logistic regression. Breast Cancer Res Treat. 2014 Feb;144(1):193-204. PMID: 24510010. doi: 10.1007/s10549-014-2850-9.
105 Maitland A, Lin A, Cantor D, Jones M, Moser RP, Hesse BW, et al. A Nonresponse Bias Analysis of the Health Information National Trends Survey (HINTS). Journal of health communication. 2017 Jul;22(7):545-53. PMID: 28557627. doi: 10.1080/10810730.2017.1324539.
106 Manganello JA, Clayman ML. The association of understanding of medical statistics with health information seeking and health provider interaction in a national sample of young adults. Journal of health communication. 2011;16 Suppl 3:163-76. PMID: 21951250. doi: 10.1080/10810730.2011.604704.
107 Marks R, Ok H, Joung H, Allegrante JP. Perceptions about collaborative decisions: Perceived provider effectiveness among 2003 and 2007 Health Information National Trends Survey (HINTS) respondents. Journal of health communication. 2010;15(Suppl 3):135-46. PMID: 2010-25748-012. doi: 10.1080/10810730.2010.522701.
108 Massey PM, Langellier BA, Sentell T, Manganello J. Nativity and language preference as drivers of health information seeking: examining differences and trends from a U.S. population-based survey. Ethn Health. 2017 Dec;22(6):596-609. PMID: 27766894. doi: 10.1080/13557858.2016.1244745.
109 Massey PM. Adoption and use of internet technologies in health communication: Examining disparities in diffusion patterns, health information sources, and patient-provider encounters. US: ProQuest Information & Learning; 2014.
110 Massey PM. Where Do U.S. Adults Who Do Not Use the Internet Get Health Information? Examining Digital Health Information Disparities From 2008 to 2013. Journal of health communication. 2016;21(1):118-24. PMID: 26166484. doi: 10.1080/10810730.2015.1058444.
111 Miller LM, Bell RA. Online health information seeking: the influence of age, information trustworthiness, and search challenges. J Aging Health. 2012 Apr;24(3):525-41. PMID: 22187092. doi: 10.1177/0898264311428167.
112 Moldovan M. Risk perceptions, worry and communication as predictors of lung, colon and skin cancer-related behaviors. US: ProQuest Information & Learning; 2010.
113 Moser RP, Arndt J, Han PK, Waters EA, Amsellem M, Hesse BW. Perceptions of cancer as a death sentence: prevalence and consequences. J Health Psychol. 2014 Dec;19(12):1518-24. PMID: 23864071. doi: 10.1177/1359105313494924.
114 Myrick JG, Willoughby JF. Educated but anxious: How emotional states and education levels combine to influence online health information seeking. Health informatics journal. 2017 Jul 01:1460458217719561. PMID: 28728457. doi: 10.1177/1460458217719561.
115 Nan X, Underhill J, Jiang H, Shen H, Kuch B. Risk, efficacy, and seeking of general, breast, and prostate cancer information. Journal of health communication. 2012;17(2):199-211. PMID: 22026448. doi: 10.1080/10810730.2011.585690.
116 Nguyen AB, Robinson J, O'Brien EK, Zhao X. Racial and Ethnic Differences in Tobacco Information Seeking and Information Sources: Findings From the 2015 Health Information National Trends Survey. Journal of health communication. 2017 Sep;22(9):743-52. PMID: 28762887. doi: 10.1080/10810730.2017.1347216.
117 Niederdeppe J, Frosch DL, Hornik RC. Cancer news coverage and information seeking. Journal of health communication. 2008 Mar;13(2):181-99. PMID: 18300068. doi: 10.1080/10810730701854110.
118 Niederdeppe J. Beyond knowledge gaps: Examining socioeconomic differences in response to cancer news. Human Communication Research. 2008;34(3):423-47. PMID: 2008-09738-004. doi: 10.1111/j.1468-2958.2008.00327.x.
119 Nogueira LM, Thai CL, Nelson W, Oh A. Nutrition Label Numeracy: Disparities and Association with Health Behaviors. Am J Health Behav. 2016 Jul;40(4):427-36. PMID: 27338989. doi: 10.5993/ajhb.40.4.4.
120 Oh YS, Song NK. Investigating Relationships Between Health-Related Problems and Online Health Information Seeking. Comput Inform Nurs. 2017 Jan;35(1):29-35. PMID: 26950091. doi: 10.1097/cin.0000000000000234.
121 Oh YS. Predictors of Self and Surrogate Online Health Information Seeking in Family Caregivers to Cancer Survivors. Soc Work Health Care. 2015;54(10):939-53. PMID: 26671245. doi: 10.1080/00981389.2015.1070780.

122 Persoskie A, Hennessy E, Nelson WL. US Consumers' Understanding of Nutrition Labels in 2013: The Importance of Health Literacy. Prev Chronic Dis. 2017 Sep 28;14:E86. PMID: 28957033. doi: 10.5888/pcd14.170066.

123 Peterson EB, Chou WS, Kelley DE, Hesse B. Trust in national health information sources in the United States: comparing predictors and levels of trust across three health domains. Transl Behav Med. 2020 Oct 8;10(4):978-88. PMID: 31116400. doi: 10.1093/tbm/ibz066.

124 Prestin A, Vieux SN, Chou WY. Is Online Health Activity Alive and Well or Flatlining? Findings From 10 Years of the Health Information National Trends Survey. Journal of health communication. 2015;20(7):790-8. PMID: 26042588. doi: 10.1080/10810730.2015.1018590.

125 Quillin JM. Lifestyle Risk Factors Among People Who Have Had Cancer Genetic Testing. Journal of genetic counseling. 2016 Oct;25(5):957-64. PMID: 26659117. doi: 10.1007/s10897-015-9925-6.

126 Rains SA. Health at high speed: Broadband internet access, health communication, and the digital divide. Communication Research. 2008;35(3):283-97. PMID: 2008-06361-001. doi: 10.1177/0093650208315958.

127 Richardson A, Allen JA, Xiao H, Vallone D. Effects of race/ethnicity and socioeconomic status on health information-seeking, confidence, and trust. J Health Care Poor Underserved. 2012 Nov;23(4):1477-93. PMID: 23698662. doi: 10.1353/hpu.2012.0181.

128 Roach AR, Lykins EL, Gochett CG, Brechting EH, Graue LO, Andrykowski MA. Differences in cancer information-seeking behavior, preferences, and awareness between cancer survivors and healthy controls: a national, population-based survey. J Cancer Educ. 2009;24(1):73-9. PMID: 19259869. doi: 10.1080/08858190802664784.

129 Robison-Chadwell A. US young adults STDs, risk perception, risk behaviors, and health information seeking: ProQuest Information & Learning; 2018.

130 Ruppel EK, Rains SA. Information sources and the health information-seeking process: An application and extension of channel complementarity theory. Communication Monographs. 2012;79(3):385-405. PMID: 2012-18221-006. doi: 10.1080/03637751.2012.697627.

131 Ruppel EK. Scanning Health Information Sources: Applying and Extending the Comprehensive Model of Information Seeking. Journal of health communication. 2016;21(2):208-16. PMID: 26716985. doi: 10.1080/10810730.2015.1058438.

132 Rutten L, Stevenson S. P04.20. Health informaton seeking, trust in information sources and use of complementary and alternative medicine. BMC Complement Altern Med. 2012;12(Suppl 1):1-. PMID: 104481657. Language: English. Entry Date: 20121129. Revision Date: 20150711. Publication Type: Journal Article. doi: 10.1186/1472-6882-12-S1-P290.

133 Rutten LJ, Augustson EM, Doran KA, Moser RP, Hesse BW. Health information seeking and media exposure among smokers: a comparison of light and intermittent tobacco users with heavy users. Nicotine & tobacco research : official journal of the Society for Research on Nicotine and Tobacco. 2009 Feb;11(2):190-6. PMID: 19264865. doi: 10.1093/ntr/ntn019.

134 Senft N, Everson J. eHealth Engagement as a Response to Negative Healthcare Experiences: Cross-Sectional Survey Analysis. J Med Internet Res. 2018 Dec 5;20(12):e11034. PMID: 30518513. doi: 10.2196/11034.

135 Shaffer KM, Chow PI, Cohn WF, Ingersoll KS, Ritterband LM. Informal Caregivers' Use of Internet-Based Health Resources: An Analysis of the Health Information National Trends Survey. JMIR Aging. 2018 Dec 18;1(2):e11051. PMID: 31518244. doi: 10.2196/11051.

136 Shahab L, Brown J, Gardner B, Smith SG. Seeking health information and support online: does it differ as a function of engagement in risky health behaviors? Evidence from the health information national trends survey. J Med Internet Res. 2014 Nov 06;16(11):e253. PMID: 25380308. doi: 10.2196/jmir.3368.

137 Shea-Budgell MA, Kostaras X, Myhill KP, Hagen NA. Information needs and sources of information for patients during cancer follow-up. Curr Oncol. 2014 Aug;21(4):165-73. PMID: 25089098. doi: 10.3747/co.21.1932.

138 Shen H, Xu J, Wang Y. Applying Situational Theory of Problem Solving in Cancer Information Seeking: A Cross-Sectional Analysis of 2014 HINTS Survey. J Health Commun. 2019;24(2):165-73. PMID: 30849294. doi: 10.1080/10810730.2019.1587111.

139 Sherman LD, Patterson MS, Tomar A, Wigfall LT. Use of Digital Health Information for Health Information Seeking Among Men Living With Chronic Disease: Data From the Health Information National Trends Survey. Am J Mens Health. 2020 Jan-Feb;14(1):1557988320901377. PMID: 31973642. doi: 10.1177/1557988320901377.

140 Shim M. Connecting internet use with gaps in cancer knowledge. Health communication. 2008 Sep;23(5):448-61. PMID: 18850392. doi: 10.1080/10410230802342143.

141 Shimoga SV, Lu YZ. Role of provider encouragement on patient engagement via online portals. J Am Med Inform Assoc. 2019 Oct 1;26(10):968-76. PMID: 30925585. doi: 10.1093/jamia/ocz026.

142 Shneyderman Y, Rutten LJ, Arheart KL, Byrne MM, Kornfeld J, Schwartz SJ. Health Information Seeking and Cancer Screening Adherence Rates. J Cancer Educ. 2016 Mar;31(1):75-83. PMID: 25619195. doi: 10.1007/s13187-015-0791-6.

143 Simonovic N, Taber JM, Klein WMP, Ferrer RA. Evidence that perceptions of and tolerance for medical ambiguity are distinct constructs: An analysis of nationally representative US data. Health Expect. 2020 Jun;23(3):603-13. PMID: 32097530. doi: 10.1111/hex.13037.

144 Sinky TH, Faith J, Lindly O, Thorburn S. Cancer Fatalism and Preferred Sources of Cancer Information: an Assessment Using 2012 HINTS Data. J Cancer Educ. 2016 Sep 20. PMID: 27650861. doi: 10.1007/s13187-016-1115-1.

145 Smith SG, Wolf MS, von Wagner C. Socioeconomic status, statistical confidence, and patient-provider communication: an analysis of the Health Information National Trends Survey (HINTS 2007). Journal of health communication. 2010;15 Suppl 3:169-85. PMID: 21154092. doi: 10.1080/10810730.2010.522690.

146 Soo Jung H, Kyung Han Y. The Effects of Experienced Uncertainty and Patients' Assessments of Cancer-Related Information-Seeking Experiences on Fatalistic Beliefs and Trust in Physicians. Health communication. 2016;31(12):1495-505. PMID: 118088914. Language: English. Entry Date: 20160922. Revision Date: 20161011. Publication Type: Article. Journal Subset: Blind Peer Reviewed. doi: 10.1080/10410236.2015.1089399.

147 Soto-Perez-de-Celis E, Perez-Montessoro V, Rojo-Castillo P, Chavarri-Guerra Y. Health-Related Information-Seeking Behaviors and Preferences Among Mexican Patients with Cancer. J Cancer Educ. 2018 Jun;33(3):505-9. PMID: 29442291. doi: 10.1007/s13187-018-1334-8.

148 Spleen AM, Lengerich EJ, Camacho FT, Vanderpool RC. Health care avoidance among rural populations: results from a nationally representative survey. J Rural Health. 2014 Winter;30(1):79-88. PMID: 24383487. doi: 10.1111/jrh.12032.

149 Spooner KK, Salemi JL, Salihu HM, Zoorob RJ. eHealth patient-provider communication in the United States: interest, inequalities, and predictors. J Am Med Inform Assoc. 2017 Apr 1;24(e1):e18-e27. PMID: 27497797. doi: 10.1093/jamia/ocw087.

150 Strekalova YA. Electronic health record use among cancer patients: Insights from the Health Information National Trends Survey. Health informatics journal. 2017 Apr 01:1460458217704246. PMID: 28434278. doi: 10.1177/1460458217704246.

151 Sun M, Jiang LC. Interpersonal influences on self-management in the eHealth era: Predicting the uses of eHealth tools for self-care in America. Health Soc Care Community. 2020 Jul 27. PMID: 32716139. doi: 10.1111/hsc.13107.

152 Suziedelyte A. How does searching for health information on the Internet affect individuals' demand for health care services? Soc Sci Med. 2012 Nov;75(10):1828-35. PMID: 22884947. doi: 10.1016/j.socscimed.2012.07.022.

153 Swoboda CM, Van Hulle JM, McAlearney AS, Huerta TR. Odds of talking to healthcare providers as the initial source of healthcare information: updated cross-sectional results from the Health Information National Trends Survey (HINTS). BMC Fam Pract. 2018 Aug 29;19(1):146. PMID: 30157770. doi: 10.1186/s12875-018-0805-7.

154 Taber JM, Howell JL, Emanuel AS, Klein WM, Ferrer RA, Harris PR. Associations of spontaneous self-affirmation with health care experiences and health information seeking in a national survey of US adults. Psychol Health. 2016;31(3):292-309. PMID: 26315683. doi: 10.1080/08870446.2015.1085986.

155 Taber JM, Klein WM, Ferrer RA, Kent EE, Harris PR. Optimism and Spontaneous Self-affirmation are Associated with Lower Likelihood of Cognitive Impairment and Greater Positive Affect among Cancer Survivors. Ann Behav Med. 2016 Apr;50(2):198-209. PMID: 26497697. doi: 10.1007/s12160-015-9745-9.

156 Thomas KB, Simpson SL, Tarver WL, Gwede CK. Is social support from family associated with PSA testing? An exploratory analysis using the Health Information National Trends Survey (HINTS) 2005. American journal of men's health. 2010 Mar;4(1):50-9. PMID: 19477731. doi: 10.1177/1557988308328541.

157 Thompson EL, Wheldon CW, Vamos CA, Griner SB, Daley EM. How Is Health Literacy Related to Pap Testing Among US Women? J Cancer Educ. 2019 Aug;34(4):789-95. PMID: 29931455. doi: 10.1007/s13187-018-1375-z.

158 Tian Y, Robinson JD. Incidental health information use and media complementarity: a comparison of senior and non-senior cancer patients. Patient Educ Couns. 2008 Jun;71(3):340-4. PMID: 18372141. doi: 10.1016/j.pec.2008.02.006.

159 Tian Y, Robinson JD. Incidental health information use on the Internet. Health communication. 2009 Jan;24(1):41-9. PMID: 19204857. doi: 10.1080/10410230802606984.

160 Tian Y, Robinson JD. Media complementarity and health information seeking in Puerto Rico. Journal of health communication. 2014;19(6):710-20. PMID: 24377383. doi: 10.1080/10810730.2013.821558.

161 Tian Y, Robinson JD. Media use and health information seeking: an empirical test of complementarity theory. Health communication. 2008 Mar-Apr;23(2):184-90. PMID: 18444004. doi: 10.1080/10410230801968260.

162 Tortolero-Luna G, Finney Rutten LJ, Hesse BW, Davis T, Kornfeld J, Sanchez M, et al. Health and cancer information seeking practices and preferences in Puerto Rico: creating an evidence base for cancer communication efforts. Journal of health communication. 2010;15 Suppl 3:30-45. PMID: 21154082. doi: 10.1080/10810730.2010.522698.

163 Upadhyay S, Lord J, Gakh M. Health-Information Seeking and Intention to Quit Smoking: Do Health Beliefs Have a Mediating Role? Tob Use Insights. 2019;12:1179173x19871310. PMID: 31488954. doi: 10.1177/1179173x19871310.

164 Valle CG, Tate DF, Mayer DK, Allicock M, Cai J, Campbell MK. Physical activity in young adults: A signal detection analysis of Health Information National Trends Survey (HINTS) 2007 data. Journal of health communication. 2015;20(2):134-46. PMID: 2015-09988-003. doi: 10.1080/10810730.2014.917745.

165 Van Stee SK, Yang Q. Online Cancer Information Seeking: Applying and Extending the Comprehensive Model of Information Seeking. Health communication. 2017 Oct 30:1-10. PMID: 29083231. doi: 10.1080/10410236.2017.1384350.

166 Vanderpool RC, Huang B. Cancer risk perceptions, beliefs, and physician avoidance in Appalachia: Results from the 2008 HINTS Survey. Journal of health communication. 2010;15(Suppl 3):78-91. PMID: 2010-25748-008. doi: 10.1080/10810730.2010.522696.

167 Viswanath K, Ackerson LK. Race, ethnicity, language, social class, and health communication inequalities: a nationally-representative cross-sectional study. PLoS One. 2011 Jan 18;6(1):e14550. PMID: 21267450. doi: 10.1371/journal.pone.0014550.

168 Volkman JE, Luger TM, Harvey KL, Hogan TP, Shimada SL, Amante D, et al. The National Cancer Institute's Health Information National Trends Survey [HINTS]: a national cross-sectional analysis of talking to your doctor and other healthcare providers for health information. BMC Fam Pract. 2014 Jun 06;15:111. PMID: 24906558. doi: 10.1186/1471-2296-15-111.

169 Waters EA, Wheeler C, Hamilton JG. How Are Information Seeking, Scanning, and Processing Related to Beliefs About the Roles of Genetics and Behavior in Cancer Causation? Journal of health communication. 2016;21(sup2):6-15. PMID: 27661291. doi: 10.1080/10810730.2016.1193917.

170 Wheldon CW, Kaufman AR, Moser RP, Hoffman L, Keely O'Brien E, Delahanty J, et al. The role of sexual identity in tobacco information–seeking behaviours and perceptions. Health Education Journal. 2019;78(2):203-13. PMID: 135372820. Language: English. Entry Date: 20190319. Revision Date: 20190417. Publication Type: Article. doi: 10.1177/0017896918801391.

171 Wigfall LT, Friedman DB. Cancer Information Seeking and Cancer-Related Health Outcomes: A Scoping Review of the Health Information National Trends Survey Literature. Journal of health communication. 2016 Sep;21(9):989-1005. PMID: 27466828. doi: 10.1080/10810730.2016.1184358.

172 Wigfall LT, Tanner AH. Health Literacy and Health-Care Engagement as Predictors of Shared Decision-Making Among Adult Information Seekers in the USA: a Secondary Data Analysis of the Health Information National Trends Survey. J Cancer Educ. 2016 Jun 02. PMID: 27251634. doi: 10.1007/s13187-016-1052-z.

173 Wiseman KP, Klein WMP. Evaluating Correlates of Awareness of the Association between Drinking Too Much Alcohol and Cancer Risk in the United States. Cancer Epidemiol Biomarkers Prev. 2019 Jul;28(7):1195-201. PMID: 31043419. doi: 10.1158/1055-9965.Epi-18-1010.

174 Wiseman KP, Margolis KA, Bernat JK, Grana RA. The association between perceived e-cigarette and nicotine addictiveness, information-seeking, and e-cigarette trial among U.S. adults. Prev Med. 2019 Jan;118:66-72. PMID: 30315847. doi: 10.1016/j.ypmed.2018.10.003.

175 Wong KY, Do YK. Are there socioeconomic disparities in women having discussions on human papillomavirus vaccine with health care providers? BMC Womens Health. 2012 Oct 04;12:33. PMID: 23033931. doi: 10.1186/1472-6874-12-33.

176 Wu QL, Street RL, Jr. Factors affecting cancer patients' electronic communication with providers: Implications for COVID-19 induced transitions to telehealth. Patient Educ Couns. 2020 Sep 28;103(12):2583-7. PMID: 33036815. doi: 10.1016/j.pec.2020.09.036.

177 Xiang J, Stanley SJ. From online to offline: Exploring the role of e-health consumption, patient involvement, and patient-centered communication on perceptions of health care quality. Comput Human Behav. 2017;70:446-52. PMID: 121619437. Language: English. Entry Date: 20170313. Revision Date: 20170313. Publication Type: Article. Journal Subset: Biomedical. doi: 10.1016/j.chb.2016.12.072.

178 Xiao Z, Lee J, Zeng L, Ni L. Information seeking in the context of cigarette smoking: predictors from the Comprehensive Model of Information Seeking (CMIS). Psychol Health Med. 2020 Dec;25(10):1228-46. PMID: 32077752. doi: 10.1080/13548506.2020.1728348.

179 Ye J, Shim R. Perceptions of health care communication: examining the role of patients' psychological distress. J Natl Med Assoc. 2010 Dec;102(12):1237-42. PMID: 21287905.

180 Ye J, Williams SD, Xu Z. The association between social networks and colorectal cancer screening in American males and females: data from the 2005 Health Information National Trends Survey. Cancer Causes Control. 2009 Sep;20(7):1227-33. PMID: 19350400. doi: 10.1007/s10552-009-9335-x.

181 Ye J, Xu Z, Aladesanmi O. Provider recommendation for colorectal cancer screening: examining the role of patients' socioeconomic status and health insurance. Cancer Epidemiol. 2009 Oct;33(3-4):207-11. PMID: 19716780. doi: 10.1016/j.canep.2009.07.011.

182 Ye Y. A path analysis on correlates of consumer trust in online health information: evidence from the health information national trends survey. Journal of health communication. 2010;15 Suppl 3:200-15. PMID: 21154094. doi: 10.1080/10810730.2010.522687.

183 Ye Y. Correlates of consumer trust in online health information: findings from the health information national trends survey. Journal of health communication. 2011 Jan;16(1):34-49. PMID: 21086209. doi: 10.1080/10810730.2010.529491.

184 Yeo Y. Indirect effects of contextual factors on patients' consultations with healthcare professionals about health information found online. BMC Health Serv Res. 2016 Aug 30;16:447. PMID: 27576443. doi: 10.1186/s12913-016-1713-y.

185 Zahnd WE, Goldfarb J, Scaife SL, Francis ML. Rural-urban differences in behaviors to prevent skin cancer: an analysis of the Health Information National Trends Survey. J Am Acad Dermatol. 2010 Jun;62(6):950-6. PMID: 20236728. doi: 10.1016/j.jaad.2009.08.058.

186 Zhang Y, Sun Y, Kim Y. The influence of individual differences on consumer's selection of online sources for health information. Computers in Human Behavior. 2017;67:303-12. PMID: 119965559. Language: English. Entry Date: 20161209. Revision Date: 20161209. Publication Type: Article. doi: 10.1016/j.chb.2016.11.008.

187 Zhao X, Cai X. The role of risk, efficacy, and anxiety in smokers' cancer information seeking. Health communication. 2009 Apr;24(3):259-69. PMID: 19415558. doi: 10.1080/10410230902805932.

188 Zhao X, Yang B, Wong CW. Analyzing Trend for U.S. Immigrants' e-Health Engagement from 2008 to 2013. Health Commun. 2019 Oct;34(11):1259-69. PMID: 29768069. doi: 10.1080/10410236.2018.1475999.

2. US_Health Tracking Survey (Pew Research Center)

1 Ardito SC. Seeking Consumer Health Information on the Internet. Online Searcher. 2013;37(4):45-8. PMID: 107969921. Language: English. Entry Date: 20130904. Revision Date: 20150712. Publication Type: Journal Article.

2 Chisolm DJ, Sarkar M. E-health use in african american internet users: can new tools address old disparities? Telemed J E Health. 2015 Mar;21(3):163-9. PMID: 25536065. doi: 10.1089/tmj.2014.0107.

3 Chisolm DJ. Does online health information seeking act like a health behavior? A test of the behavioral model. Telemedicine and e-Health. 2010;16(2):154-60. PMID: 2010-06243-002. doi: 10.1089/tmj.2009.0102.

4 Kuehn BM. More than one-third of US individuals use the Internet to self-diagnose. JAMA: Journal of the American Medical Association. 2013;309(8):756-7. PMID: 2013-14743-009. doi: 10.1001/jama.2013.629.

5 Oh YS, Cho Y. Examining the relationships between resources and online health information seeking among patients with chronic diseases and healthy people. Soc Work Health Care. 2015;54(2):83-100. PMID: 2015-06969-001. doi: 10.1080/00981389.2014.987940.

6 Ruggiero KJ, Gros DF, McCauley J, de Arellano MA, Danielson CK. Rural adults' use of health-related information online: Data from a 2006 national online health survey. Telemedicine and e-Health. 2011;17(5):329-34. PMID: 2011-13562-003. doi: 10.1089/tmj.2010.0195.

7 Saulsberry L, Price M, Hsu J. Insurance coverage & Whither Thou Goest for health information in 2012. Medicare & medicaid research review. 2014;4(4). PMID: 25383242. doi: 10.5600/mmrr.004.04.b01.

8 Stern MJ, Cotten SR, Drentea P. The separate spheres of online health: Gender, parenting, and online health information searching in the information age. Journal of Family Issues. 2012;33(10):1324-50. PMID: 2012-26443-002. doi: 10.1177/0192513X11425459.

9 Sun Y, Liu M, Krakow M. Health e-mavens: identifying active online health information users. Health Expect. 2016 Oct;19(5):1071-83. PMID: 26296041. doi: 10.1111/hex.12398.

3. US_Annenberg National Health Communication Survey (ANHCS) (Annenberg Schools for communication at the University of Pennsylvania & the University of Southern California)

1 Bigsby E, Hovick SR. Understanding Associations between Information Seeking and Scanning and Health Risk Behaviors: An Early Test of the Structural Influence Model. Health Commun. 2018 Mar;33(3):315-25. PMID: 28059570. doi: 10.1080/10410236.2016.1266575.

2 Hovick SR, Bigsby E. Heart Disease and Colon Cancer Prevention Beliefs and Their Association With Information Seeking and Scanning. Journal of health communication. 2016;21(1):76-84. PMID: 26444664. doi: 10.1080/10810730.2015.1049307.

3 Ishikawa Y, Kondo N, Kawachi I, Viswanath K. Are socioeconomic disparities in health behavior mediated by differential media use? Test of the communication inequality theory. Patient Educ Couns. 2016 Nov;99(11):1803-7. PMID: 27349600. doi: 10.1016/j.pec.2016.05.018.

4 Kim S. An exploratory study of inactive health information seekers. Int J Med Inform. 2015 Feb;84(2):119-33. PMID: 25453277. doi: 10.1016/j.ijmedinf.2014.10.003.

5 Lee C-J. Does the Internet displace health professionals? Journal of health communication. 2008;13(5):450-64. PMID: 2008-10646-003. doi: 10.1080/10810730802198839.

6 Li W, Watts J, Tan N. From Screen to Screening: Entertainment and News Television Media Effects on Cancer Screening Behaviors. J Health Commun. 2019;24(4):385-94. PMID: 31033423. doi: 10.1080/10810730.2019.1607954.

7 Yang Q, Chen Y, Wendorf Muhamad J. Social Support, Trust in Health Information, and Health Information-Seeking Behaviors (HISBs): A Study Using the 2012 Annenberg National Health Communication Survey (ANHCS). Health communication. 2017 Sep;32(9):1142-50. PMID: 27589249. doi: 10.1080/10410236.2016.1214220.

4. US_National Health Interview Survey (NHIS) (National Center for Health Statistics)

1 Amante DJ, Hogan TP, Pagoto SL, English TM, Lapane KL. Access to care and use of the Internet to search for health information: results from the US National Health Interview Survey. J Med Internet Res. 2015 Apr 29;17(4):e106. PMID: 25925943. doi: 10.2196/jmir.4126.

2 Dahlhamer JM, Galinsky AM, Joestl SS, Ward BW. Sexual Orientation and Health Information Technology Use: A Nationally Representative Study of U.S. Adults. LGBT health. 2017 Apr;4(2):121-9. PMID: 28287875. doi: 10.1089/lgbt.2016.0199.

3 Gonzalez M, Sanders-Jackson A, Wright T. Web-Based Health Information Technology: Access Among Latinos Varies by Subgroup Affiliation. J Med Internet Res. 2019 Apr 16;21(4):e10389. PMID: 30990462. doi: 10.2196/10389.

4 Heckman CJ, Handorf E, Auerbach MV. Prevalence and Correlates of Skin Cancer Screening Among Indoor Tanners and Nontanners. JAMA Dermatol. 2018 May 1;154(5):554-60. PMID: 29617518. doi: 10.1001/jamadermatol.2018.0163.

5 Hong Y-R, Hincapie-Castillo JM, Xie Z, Segal R, Mainous Iii AG. Socioeconomic and Demographic Characteristics of US Adults Who Purchase Prescription Drugs From Other Countries. JAMA Network Open. 2020;3(6):e208968-e. PMID: 144266662. Language: English. Entry Date: 20200703. Revision Date: 20200724. Publication Type: Article. doi: 10.1001/jamanetworkopen.2020.8968.

6 Kindratt T, Callender L, Cobbaert M, Wondrack J, Bandiera F, Salvo D. Health information technology use and influenza vaccine uptake among US adults. Int J Med Inform. 2019 Sep;129:37-42. PMID: 31445279. doi: 10.1016/j.ijmedinf.2019.05.025.

7 Lee JH, Giovenco D, Operario D. Patterns of Health Information Technology Use according to Sexual Orientation among US Adults Aged 50 and Older: Findings from a National Representative Sample-National Health Interview Survey 2013-2014. Journal of health communication. 2017 Aug;22(8):666-71. PMID: 28749748. doi: 10.1080/10810730.2017.1341566.

8 Mahajan S, Lu Y, Spatz ES, Nasir K, Krumholz HM. Trends and Predictors of Use of Digital Health Technology in the United States. Am J Med. 2021 Jan;134(1):129-34. PMID: 32717188. doi: 10.1016/j.amjmed.2020.06.033.

9 Us Centers For Disease C, Prevention Epilepsy P. Internet use and looking up information online in adults with epilepsy varies by epilepsy status--2013 National Health Interview Survey. Epilepsy Behav. 2016 Jan;54:47-9. PMID: 26655448. doi: 10.1016/j.yebeh.2015.10.019.

10 Yin R, Neyens DM. Online Health Resource Use by Individuals With Inflammatory Bowel Disease: Analysis Using the National Health Interview Survey. J Med Internet Res. 2020 Sep 24;22(9):e15352. PMID: 32969831. doi: 10.2196/15352.

11 Zhang Y, Lauche R, Sibbritt D, Olaniran B, Cook R, Adams J. Comparison of Health Information Technology Use Between American Adults With and Without Chronic Health Conditions: Findings From The National Health Interview Survey 2012. J Med Internet Res. 2017 Oct 05;19(10):e335. PMID: 28982644. doi: 10.2196/jmir.6989.

5. US_Health Tracking Household Survey (HTHS) (Center for Studying Health System Change (HSC))

1 Dean CA, Geneus CJ, Rice S, Johns M, Quasie-Woode D, Broom K, et al. Assessing the significance of health information seeking in chronic condition management. Patient Educ Couns. 2017 Aug;100(8):1519-26. PMID: 28320559. doi: 10.1016/j.pec.2017.03.012.

2 Rooks RN, Kapral CG, Mathis AL. Chronic Conditions May Be More Important Than Race or Ethnicity in Relation to Health Information Seeking and Use. J Aging Health. 2019 Apr;31(4):611-30. PMID: 29254425. doi: 10.1177/0898264317744643.

3 Rooks RN, Wiltshire JC, Elder K, BeLue R, Gary LC. Health information seeking and use outside of the medical encounter: is it associated with race and ethnicity? Soc Sci Med. 2012 Jan;74(2):176-84. PMID: 22154611. doi: 10.1016/j.socscimed.2011.09.040.

4 Wiltshire JC, Roberts V, Brown R, Sarto GE. The effects of socioeconomic status on participation in care among middle-aged and older adults. J Aging Health. 2009 Mar;21(2):314-35. PMID: 19091692. doi: 10.1177/0898264308329000.

6. Europe_Flash Eurobarometer 404 (European citizen's digital health literacy) (European Commission)

1 Reifegerste D, Bachl M, Baumann E. Surrogate health information seeking in Europe: Influence of source type and social network variables. Int J Med Inform. 2017 Jul;103:7-14. PMID: 28551004. doi: 10.1016/j.ijmedinf.2017.04.006.

7. France_French Health Barometer (Baromètre santé) (National Institute for Prevention and Health Education (Institut national de préventior

1 Beck F, Richard JB, Nguyen-Thanh V, Montagni I, Parizot I, Renahy E. Use of the internet as a health information resource among French young adults: results from a nationally representative survey. J Med Internet Res. 2014 May 13;16(5):e128. PMID: 24824164. doi: 10.2196/jmir.2934.

8. Germany_Gesundheitsmonitor (Bertelsmann Foundation in cooperation with the Barmer GEK, a statutory health insurance)

1 Baumann E, Czerwinski F, Reifegerste D. Gender-Specific Determinants and Patterns of Online Health Information Seeking: Results From a Representative German Health Survey. J Med Internet Res. 2017 Apr 04;19(4):e92. PMID: 28377367. doi: 10.2196/jmir.6668.

9. Germany_Stiftung Gesundheitswissen (HINTS Germany) / GESIS Institute (Mannheim / Cologne) and the Robert Koch Institute (RKI, Berlin)

1 Baumann E, Czerwinski F, Rosset M, Seelig M, Suhr R. How do people in Germany seek health information? Insights from the first wave of HINTS Germany. Bundesgesundheitsblatt Gesundheitsforschung Gesundheitsschutz. 2020 Sep;63(9):1151-60. PMID: 32666180. doi: 10.1007/s00103-020-03192-x.

10. Israel_(Not titled) (Individual researchers funded by Israel's Ministry of Science, Technology & Space)

1 Hayat TZ, Brainin E, Neter E. With Some Help From My Network: Supplementing eHealth Literacy With Social Ties. J Med Internet Res. 2017 Mar 30;19(3):e98. PMID: 28360024. doi: 10.2196/jmir.6472.

11. Poland_E-Health Consumer Trend Survey 2012 (Individual researcher (Wroclaw Medical University))

1 Bujnowska-Fedak MM. Trends in the use of the Internet for health purposes in Poland. BMC Public Health. 2015 Feb 27;15:194. PMID: 25886280. doi: 10.1186/s12889-015-1473-3.

12. South Korea_Survey of cancer and health related information seeking behavior for Korean (Individual researcher funded by the National Research Foundation of Korea)

1 Hanna Choi. Classification of health information-seeking behavior among Korean adults The 23rd East Asian Forum of Nursing Scholars (EAFONS); Jan, 2020; Chiang Mai, Thailand.

13. Taiwan_Taiwan Communication Survey (TCS) (the Ministry and Science Technology)

1 Chang C. Self-Control-Centered Empowerment Model: Health Consciousness and Health Knowledge as Drivers of Empowerment-Seeking through Health Communication. Health Communication. 2020;35(12):1497-508. PMID: 145890315. Language: English. Entry Date: 20200924. Revision Date: 20200930. Publication Type: Article. doi: 10.1080/10410236.2019.1652385.
