# Supplementary material for: Characteristics of the Measurement Tools for Assessing Health Information–Seeking Behaviors in Nationally Representative Surveys: Systematic Review
Source: J Med Internet Res. 2021 Jul 26;23(7):e27539. doi: 10.2196/27539 (PMC8367171; doi:10.2196/27539)
Supplement: Multimedia Appendix 3 [file jmir_v23i7e27539_app3.pdf]

Multimedia Appendix 3. Characteristics of the health information seeking instruments

#	Country	Language	Instrument	Recent survey version	Administration Institution	Purpose of the measurement	Frequency	Survey years	Sampling method	Mode of administration	Target population	Total no. of population of the recent survey
1	USA	English, Spanish	Health Information National Trends Survey (HINTS)	2019, HINTS 5, Cycle 3	National Cancer Institute (NCI)	To investigate respondents' access to and use of health information, including information technology to manage health and health information	Every few years (1-2 year cycle)	2003-present	Stratified sample of addresses and a selected adult within the random sampled household	Postal mail and Web	Civilian noninstitutionalized adults aged 18 or older	N= 5247
2	USA	English, Spanish	Health Tracking Survey	2012	Pew Research Center	To assess pursuit of health taking place within a widening network of both online and offline sources	Irregular	2000-2012	Random-digit-dialing (RDD)	Telephone (interview)	Adults, aged 18 or older	N= 3014
3	USA	English	Annenberg National Health Communication Survey (ANHCS)	2012	Annenberg Schools for communication at the University of Pennsylvania and the University of Southern California	To capture national trends relating health behavior and behavioral intentions to media exposure, health knowledge and beliefs, and policy preferences and beliefs	One-cycle survey	2005-2012	RDD from an online panel (Knowledge Network Panel)	Online administration	Adults, aged 18 or older	N=3692
4	USA	English	National Health Interview Survey (NHIS)	2020	National Center for Health Statistics	To monitor the health of the population through the collection and analysis of the data	Annual	1957-present	RDD	Computer-assisted personal interviewing (CAPI)	Household	N= 33,138[a]
5	USA	English, Spanish	Health Tracking Household Survey (HTHS)	2010	Center for Studying Health System Change (HSC)	To inform health care decision makers about changes in the health care system and the influence	Irregular (2 to 5 year-period)	2010–2011	RDD	Computer assisted telephone interviewing (CATI)	Household	N=16,671 individuals (N= 9165 Family Insurance Units)
6	Europe	Mother tongue of participants	Flash Eurobarometer 404 (European citizen's digital health literacy)	2014	European Commission	To support increasing citizen's digital health to help manage their own health	One-cycle survey	2014	RDD	Telephone (interview)	EU residents aged 15 and older	N= 26,566 (28 EU countries)
7	France	French	French Health Barometer (Baromètre santé)	2017	National Institute for Prevention and Health Education (Institut national de prévention et d'éducation pour la santé)	To gain a better understanding of French health knowledge, attitudes, beliefs and behaviors.	Annual	1992-present	RDD	CATI	18-75 year old	N=15,635[b]

#	Country	Language	Instrument	Recent survey version	Administration Institution	Purpose of the measurement	Frequency	Survey years	Sampling method	Mode of administration	Target population	Total no. of population of the recent survey
8	Germany	German	Gesundheitsmonitor	2015	Bertelsmann Stiftung	To assess health-related knowledge, attitudes, and behaviors	Annual	2001-2015	Mail panel pool (GfK (German Association for Consumer Research) Mail Panel)	Postal mail	Adult (18-79 years)	N=1598
9	Germany	German	HINTS Germany	2019	Stiftung Gesundheitswissen (SGW) and the Hanover Center for Health Communication at the Institute for Journalism and Communication Research (IJK)	To close gap in important health-related information actions and yet systematical health record	Every few years (1-2 year cycle)	2018 – present	Telephone interviews	CATI	Adult (18-79 years)	N=2902
10	Israel	Hebrew, Russian, Arabic	Not titled	2014	Individual researchers funded by Israel's Ministry of Science, Technology and Space	To measure eHealth literacy for others including perceived outcome of Internet use	One-cycle survey	2014	RDD	CATI	Adult (aged 21 years and older)	N=819
11	Poland	Polish	eHealth Consumer Trend Survey 2012[c]	2012	Individual researcher (Wroclaw Medical University)	To show trend in perceptions and preferences of Polish citizens regarding Internet use and factors affecting their usage	Irregular	2005, 2007, 2012	RDD	CATI	Adults (aged 15–80+ years)	N=1000
12	South Korea	Korean	Survey of cancer and health related information seeking behavior for Korean	2018	Individual researcher funded by the National Research Foundation of Korea (NRF)	To capture national phenomenon of the cancer and health related health information seeking behavior of Korean	One-cycle survey	2018	Quota stratified random sampling	Web, Face to face survey	Adults (aged 18–65+ years)	N=1012
13	Taiwan	Mandarin Chinese	Taiwan Communication Survey (TCS)	2016	Ministry and Science Technology in Taiwan	To explores media use behaviors among the general public including health, risk, and disaster communication	Annual	1993 – 2019[d]	Probability-proportional-to-size (PPS)	Interviews	Adults (aged 18 years and older)	N=2098

[a] NHIS: 2019 sample size was reported. Data and report for 2020 NHIS will be published in fall 2021.
[b] French Health Barometer: the survey questionnaires were changed according to the survey years. The 2017 version of the survey contains HISB and is included in the study.
[c] E-Health consumer trend survey of 2012 was modified from the e-Health Consumer Trends Survey (2007), which was conducted in Denmark, Germany, Greece, Latvia, Norway, Poland, and Portugal in the WHO/European e-Health Consumer Trends project
[d] TCS: the survey questionnaires were changed according to survey year. The version of the 2016 survey contains HISB and is included in the study
