# Supplementary material for: Characteristics of the Measurement Tools for Assessing Health Information–Seeking Behaviors in Nationally Representative Surveys: Systematic Review
Source: J Med Internet Res. 2021 Jul 26;23(7):e27539. doi: 10.2196/27539 (PMC8367171; doi:10.2196/27539)
Supplement: Multimedia Appendix 4 [file jmir_v23i7e27539_app4.pdf]

Multimedia Appendix 4. Theme Occurrence Table

Domain	Subdomain		Theme	1	2	3	4	5	6	7	8	9	10	11	12	13	Count (No.)	Percentage (%)
				HINTS[a]	HTS[b]	ANHCS[c]	NHIS[d]	HTHS[e]	Europe[f]	France[g]	Germany[h]	HINTS Germany[i]	Israel[j]	Poland[k]	South Korea[l]	Taiwan[m]		
Information	Information about Health	Attitude	1. Perceived ease of use	✓		✓						✓			✓		4	30.8
			2. Perceived efficacy of seeking	✓								✓			✓		3	23.1
		Behavior	1. Seek experience (frequency)	✓		✓			✓			✓			✓	✓	6	46.2
			2. Information source	✓	✓	✓		✓		✓		✓		✓	✓		8	61.5
			3. Type of information contents	✓	✓	✓			✓	✓		✓			✓		7	53.8
			4. Purpose of search (whom for)	✓	✓	✓		✓	✓			✓			✓		7	53.8
	Patient Medical Record	Attitude	1. Perceived privacy and confidentiality risk	✓							✓				✓		3	23.1
			2. Perceived ease of use	✓											✓		2	15.4
			3. Perceived usefulness	✓							✓				✓		3	23.1
			4. Intention to use								✓			✓			2	15.4
			5. Provision of access to others	✓							✓			✓	✓		4	30.8
		Behavior	1. Access frequency	✓		✓								✓	✓		4	30.8
			2. Type of information contents sought	✓				✓							✓		3	23.1
			3. Purpose of seeking a record	✓		✓		✓							✓		4	30.8
Channel	Offline	Attitude	1. Perceived credibility	✓		✓					✓	✓			✓		5	38.5
			2. Perceived ease of use	✓				✓			✓	✓			✓		5	38.5
			3. Satisfaction with service quality	✓		✓		✓			✓	✓			✓		6	46.2
		Behavior	1. Access frequency	✓	✓	✓	✓	✓	✓	✓		✓		✓	✓	✓	11	84.6
			2. Type of health service			✓	✓	✓	✓		✓			✓			6	46.2
			3. Communication with healthcare provider (HCP)	✓	✓			✓			✓	✓			✓		6	46.2
			4. Health related decision-making	✓	✓	✓		✓			✓	✓			✓		7	53.8
	Online	Attitude	1. Perceived credibility	✓		✓			✓	✓		✓			✓	✓	7	53.8
			2. Perceived ease of use	✓		✓			✓				✓		✓		5	38.5
			3. Perceived usefulness	✓	✓			✓	✓	✓		✓			✓		7	53.8
			4. Perceived eHealth literacy (technology efficacy)	✓					✓		✓		✓	✓	✓	✓	7	53.8
			5. Satisfaction of online information					✓	✓								2	15.4
			6. Perceived confidentiality risks	✓							✓			✓	✓		4	30.8
			7. Intention to use						✓		✓			✓	✓		4	30.8
		Behavior	1. Access frequency	✓	✓	✓	✓	✓	✓	✓		✓	✓	✓	✓	✓	12	92.3
			2. Type of IT device	✓	✓	✓					✓	✓		✓	✓	✓	8	61.5
			3. Health related Web and app (software use)	✓	✓	✓					✓	✓			✓		6	46.2
			4. online resource(governmental website, Wikipedia etc)	✓	✓	✓			✓	✓		✓			✓		7	53.8
			5. Communication (consult) with HCP	✓	✓			✓	✓	✓	✓	✓	✓	✓	✓		10	76.9
			6. Communication with friends and others (Social meda, forum etc)	✓	✓				✓	✓		✓		✓	✓	✓	8	61.5
			7. Health related decision-making	✓	✓			✓	✓			✓	✓	✓	✓	✓	9	69.2
			8. Tracking /managing health state (blood test, BP f/u)	✓	✓							✓		✓	✓		5	38.5
			9. Improvement of health knowledge		✓			✓					✓	✓			4	30.8
Health	Overall health	Attitude	1. Perceived health efficacy	✓		✓		✓				✓			✓		5	38.5
			2. Concerns & belief about health			✓		✓							✓	✓	4	30.8
		Behavior	1. General health state	✓	✓	✓	✓	✓	✓	✓	✓	✓		✓	✓		11	84.6
			2. Diseases diagnosed	✓	✓	✓	✓	✓	✓		✓	✓			✓		9	69.2
			3. Height	✓		✓	✓			✓	✓	✓			✓	✓	8	61.5
			4. Weight	✓		✓	✓			✓	✓	✓			✓	✓	8	61.5
			5. Mental health	✓		✓	✓	✓		✓		✓			✓		7	53.8
			6. Caregiving	✓	✓	✓									✓		4	30.8
			7. Social support	✓		✓				✓	✓	✓			✓	✓	7	53.8
	Lifestyle	Attitude	1. Perception about nutrition			✓											1	7.7
			2. Perception about physical activity	✓		✓											2	15.4
			3. Perception about alcohol	✓						✓					✓		3	23.1
			4. Perception about tobacco	✓						✓					✓		3	23.1
		Behavior	1.Nutrition	✓		✓					✓				✓		4	30.8
			2. Physical activity	✓		✓	✓		✓	✓	✓	✓			✓		8	61.5
			3. Alcohol	✓		✓	✓			✓	✓	✓			✓		7	53.8

Domain	Subdomain	Theme		1	2	3	4	5	6	7	8	9	10	11	12	13	Count (No.)	Percentage (%)
				HINTS[a]	HTS[b]	ANHCS[c]	NHIS[d]	HTHS[e]	Europe[f]	France[g]	Germany[h]	HINTS Germany[i]	Israel[j]	Poland[k]	South Korea[l]	Taiwan[m]		
			4. Tobacco	✓		✓	✓			✓	✓	✓			✓		7	53.8
	Cancer	Attitu	1. Perception about Cancer	✓		✓					✓	✓			✓		5	38.5
		Behavior	1. Cancer check-up	✓		✓					✓	✓			✓		5	38.5
			2. Cancer diagnosed	✓	✓	✓	✓	✓			✓	✓			✓		8	61.5
Person	General characteristics		1. Age	✓	✓	✓	✓	✓	✓	✓	✓	✓	✓	✓	✓	✓	13	100.0
			2. Sex/gender	✓	✓	✓	✓	✓	✓	✓	✓	✓	✓	✓	✓	✓	13	100.0
			3. Nationality		✓	✓	✓	✓		✓							5	38.5
			4. Race	✓	✓	✓	✓	✓					✓			✓	7	53.8
			5. Language	✓		✓		✓		✓						✓	5	38.5
			6. Education	✓	✓	✓	✓	✓	✓	✓	✓	✓	✓	✓	✓	✓	13	100.0
			7. Income	✓	✓	✓	✓	✓		✓	✓	✓			✓	✓	10	76.9
			8. Occupational status	✓	✓	✓	✓	✓	✓	✓	✓	✓			✓	✓	11	84.6
			9. Marital status	✓	✓	✓	✓	✓		✓	✓	✓			✓	✓	10	76.9
			10. Health literacy			✓			✓	✓						✓	4	30.8
			11. Health insurance	✓	✓	✓	✓	✓		✓	✓	✓			✓		9	69.2
			12. Number of family member	✓	✓	✓	✓	✓		✓	✓	✓		✓	✓		10	76.9
			13. Household with internet access			✓							✓			✓	3	23.1

[a]HINTS: Health Information National Trends Survey (2019, HINTS 5, Cycle 3)

[b]HTS: Health Tracking Survey (2012)

[c]ANHCS: Annenberg National Health Communication Survey (2012)

[d]NHIS: National Health Interview Survey (2020)

[e]HTHS: Health Tracking Household Survey (2010)

[f]Europe: Flash Eurobarometer (2014)

[g]France: Baromètre santé (2017)

[h]Germany: Gesundheitsmonitor (2015)

[i]HINTS Germany: Health Information National Trends Survey Germany (2019)

[j]Israel: Not titled (2014)

[k]Poland: E-Health Consumer Trend Survey (2012)

[l]South Korea: Survey of Cancer and Health-related Information Seeking Behavior for Korean (2018)

[m]Taiwan: Taiwan Communication Survey (2016)
